# Prelimbic cortex–associated stress sensitisation and region-specific mitochondrial adaptations across preclinical approaches relevant to PTSD and depression

**DOI:** 10.1016/j.ynstr.2026.100840

**Published:** 2026-07-30

**Authors:** Charlotte S. Rye, Laetitia H.E. Ward, Clara Velázquez-Sánchez, Jeffrey W. Dalley, Amy L. Milton

**Affiliations:** a Department of Psychology, University of Cambridge, Downing Street, Cambridge, CB2 3EB, United Kingdom; b Department of Psychiatry, University of Cambridge, Herchel Smith Building, Forvie Site, Robinson Way, Cambridge, CB2 0SZ, United Kingdom

**Keywords:** Depression, PTSD, Stress sensitisation, Mitochondria, Inflammation

## Abstract

Mitochondrial dysfunction is increasingly implicated in stress-related disorders, including post-traumatic stress disorder (PTSD) and depression, but its relationship to inflammatory signalling and circuit-level vulnerability remains unclear. Here, we investigated region-specific mitochondrial adaptations across two experimental approaches of stress in rodents: stress-enhanced fear learning (SEFL) and chronic unpredictable stress (CUS), modelling PTSD- and depression-like phenotypes, respectively. Behavioural data were analysed using a novel, data-driven clustering approach to define stress-responsive phenotypes (e.g., susceptible, resilient, depressive), alongside quantification of mitochondrial oxidative phosphorylation (OXPHOS) markers across the hippocampus, basolateral amygdala, and prefrontal cortex (prelimbic and infralimbic subregions), and circulating plasma inflammatory markers.

Hippocampal mitochondrial adaptations diverged between experimental approaches, with SEFL-susceptible animals showing broad upregulation of OXPHOS complexes, whereas CUS-depressive animals exhibited more restrictive mitochondrial alterations. In contrast, the basolateral amygdala showed convergent mitochondrial adaptations across models, with increased Complex V expression observed in vulnerable phenotypes. Within the prefrontal cortex, stress effects were regionally dissociable, with the prelimbic cortex showing SEFL-specific, phenotype-dependent mitochondrial alterations, implicating it as a putative node in stress sensitisation in PTSD-like phenotypes.

Plasma TNF-α levels were significantly elevated in a phenotype- and time-dependent manner and were associated with regional mitochondrial adaptations, consistent with a role for systemic inflammatory signalling in modulating stress-related neurobiological plasticity and energy states.

These data support a multi-level model in which behavioural phenotypes reflect underlying inflammatory–mitochondrial states distributed across hippocampal, amygdala, and prefrontal circuits, providing a potential explanatory framework linking systemic inflammatory signalling, circuit-specific mitochondrial function, and vulnerability to PTSD- and depressive-like pathology.

## Highlights

• Individual differences in stress responsivity are shaped by interacting changes in inflammation and cellular energetics.
• The basolateral amygdala is a common locus of mitochondrial pathology across experimental paradigms relevant to depression and PTSD.
• The stress sensitisation observed in PTSD-like phenotypes is associated with inflammatory and mitochondrial alterations in the prelimbic cortex.
• TNF-α may act as a dynamic regulator of plasticity in trauma-related contexts.
• PTSD-like states may represent a temporally compressed or ‘accelerated’ form of depressive pathology.

## 1 Introduction

Stress-related disorders such as post-traumatic stress disorder (PTSD) impose substantial personal and societal costs, with UK healthcare expenditure exceeding £11 billion annually (Cigna, 2019), and disability weights approaching those of major chronic illnesses (Freed et al., 2010). Over half of individuals with PTSD also meet criteria for major depressive disorder (MDD) (Rytwinski et al., 2013), and this high rate of comorbidity has raised important questions regarding traditional nosological boundaries – both in terms of symptomatology and underlying mechanisms (Moring et al., 2019; Stander et al., 2014; Zhang et al., 2022). High genetic correlation and shared inflammatory signatures raise the possibility of partially convergent pathophysiology (Maes et al., 1991; Zhang et al., 2022). Inflammation-induced impairments in neuroplasticity, neurotransmission, and blood–brain barrier integrity have been implicated in both disorders (Bauer and Teixeira, 2019; Miller, 2020; Raison et al., 2006), and mitochondrial dysfunction has emerged as a potential mechanistic bridge (Bersani et al., 2016; Peng et al., 1998). Inflammatory cytokines such as IFN-γ can disrupt mitochondrial biogenesis (Li et al., 2012), while mitochondrial stress can itself amplify inflammation through release of damage-associated molecular patterns (DAMPs) (Lushchak et al., 2023) and activation of Toll-Like Receptor 9/Nuclear Factor Kappa B (TLR9/NF-κB) and nucleotide-binding domain, leucine-rich-containing family, pyrin domain-containing-3 (NLRP3) pathways (Dmytriv et al., 2023; Kawai and Akira, 2007).

The overlap in inflammatory pathways between depression and PTSD presents an opportunity for cross-disease insights that could improve both early diagnostics and therapeutic strategies. Experimental approaches in rodents offer a tractable framework to parse these shared and dissociable processes. Here, we compare two procedures in rats – one with relevance to PTSD, and the other to depression – to disentangle convergent and divergent stress-induced mechanisms. Due to its capacity to distinguish between associative fear memory and non-associative fear sensitisation, the stress-enhanced fear learning (SEFL) procedure (Rau et al., 2005; Van Assche et al., 2022) has emerged as a prominent rodent analogue of trauma. By comparing SEFL with a chronic unpredictable stress (CUS) model with relevance to depression (Zühlsdorff et al., 2023), we aim to determine whether mitochondrial and inflammatory markers differentiate distinct stress-responsive behavioural phenotypes, and whether PTSD- and depression-relevant behavioural outcomes reflect overlapping or dissociable biological processes that may be relevant to the development of stress-related psychopathology and/or stress-induced behavioural adaptation.

A key objective of this study was to examine whether trauma timing – recent versus remote exposure – influences PTSD-relevant outcomes. As no behavioural differences were observed between recent and remote trauma groups, these cohorts were pooled for subsequent analyses; however, the initial comparison allowed us to assess whether temporal proximity to trauma produced detectable behavioural effects. We further investigated potential sex differences, given the increased prevalence of PTSD and depression in women (Tolin and Foa, 2008) and the potential influence of hormonal state on fear learning and extinction (Bangasser and Valentino, 2014; Garcia et al., 2018). Based on these findings, we anticipated that susceptibility to SEFL and CUS *may* differ between males and females.

We hypothesised that animals exhibiting the PTSD- and depression-relevant phenotypes will exhibit disruptions in mitochondrial function and elevated inflammatory signalling, reflecting shared stress-related biological vulnerability. However, it remains an empirical question whether these alterations will manifest as a common molecular signature or as distinct, model-specific profiles. We further predicted that recent versus remote trauma in SEFL will not produce differential behavioural outcomes, in line with evidence that massed shock induces rapid and enduring fear sensitisation (Perusini et al., 2016; Rau and Fanselow, 2009; Van Assche et al., 2022).

## 2 Methods and materials

### 2.1 Subjects

Subjects were 152 behaviourally naïve adult outbred Lister Hooded rats (n = 76 males; Charles River, Bicester, UK) weighing at least 190 g at the start of the experiments. Rats were group-housed in fours in a vivarium maintained at 21^°^C under a reversed 12 h light/dark cycle (lights on at 1900hrs). Water and standard lab chow were available *ad libitum* except during behavioural procedures. This study was regulated under the UK Animal (Scientific Procedures) Act 1986, amendment regulations 2012 and the EU legislation on the protection of animals used for scientific purposes (Directive, 2010/63/EU) under Project Licence PP4363352 following ethical review by the University of Cambridge Animal Welfare and Ethical Review Body (AWERB). All experimental procedures were performed during the rats’ dark period.

### 2.2 Behavioural apparatus and procedures (Fig. 1)

***Conditioning Chambers:*** Two contexts (A and B) were used during the experiment. In both contexts, the ceiling was made of clear Plexiglas. The conditioning chambers were interfaced to the FearCond software written in C++ for the Whisker control system (Cardinal and Aitken, 2010). In both contexts, a 2.8 W, 24 V white ceiling houselight illuminated the chamber during the sessions. Context A consisted of Med Associates conditioning chambers (29.5 x 32.5 × 23.5 cm; Med Associates) housed within sound- and light-attenuating cubicles. The grid floors were composed of 19 stainless-steel rods connected to a shock generator and scrambler that delivered the footshocks. Below the grid floor was a removable stainless-steel tray. The rats were transported to and from the context in an opaque plastic box with a lid in groups of four or six using an elevator. Context B consisted of Paul Fray conditioning chambers (40 x 30 × 40 cm; Paul Fray Ltd.) housed within sound- and light-attenuating cubicles in a different room to Context A. The grid floors were composed of 17 stainless-steel bars connected to a shock generator and scrambler that delivered the footshock. Below the grid floor was a removable stainless-steel tray. The rats were transported to and from the context in the same opaque plastic box in groups of four using the stairs and a different route through the animal facility. All Context B sessions were video recorded for offline quantification of freezing behaviour.

***SEFL ‘Trauma’ Session (Context A):*** 96 animals (n = 48 males) were assigned to the SEFL cohorts and underwent the refined version of SEFL procedure (Van Assche et al., 2022), Sample size was determined by reference to the previous literature using the refined SEFL procedure, which typically has n = 14 per group (Van Assche et al., 2022). Groups received either 0 or 13 0.5 mA, 0.5s electric footshocks in context A over a 130-min session, spaced by fixed and equal intervals. Half of the SEFL animals received footshocks on day 1 of the experiment (remote trauma), whilst the other half received context A ‘trauma’ on day 13 (recent trauma).

***Chronic, Unpredictable Stress (Context A):*** The remaining 56 animals (n = 28 males) were assigned to the chronic stress cohort and underwent a CUS procedure based on previous literature (Dutcher et al., 2023) but with the following refinements to allow for direct comparison with the SEFL groups: (i) rats received 0 or 13 0.5 mA, 0.5s electric footshocks in context A over a 13 day period rather than 20 footshocks over a 19-day period; (ii) subjects were exposed to the shock chamber on 13 occasions as opposed to 14-16; (iii) animals were placed inside the operant chambers for one 10-min session each day compared to one 30-min session. As in the original procedure, subjects were exposed to either 0, 1 or 2 footshocks randomly during each session, with no shocks occurring in the first 2 min. Control animals received a total of 0 shocks across the sessions. For experimental animals, the first two sessions included 1 or 2 shocks, but the other sessions were randomly allocated to give a total of 13 shocks, such that rats did not experience more than one consecutive 0-shock session. Sessions were designed such that rats undergoing the CUS procedure spent the same total time in Context A as those in the SEFL cohorts (130 min).

***Contextual Fear Conditioning:*** On day 14, all rats underwent contextual fear conditioning in Context B, receiving two 0.5 mA, 0.5s footshocks (ITI 30s) after a 3 min pre-shock period. Rats remained in the chamber for 90 s following the final footshock.

***Extinction Training:*** Rats were returned to Context B for a 30-min, non-reinforced session 24hrs after contextual fear conditioning.

***Retention Test:*** Fear memory retention was tested 24hrs after extinction training with an 8-min session conducted in Context B. No footshocks were delivered.

### 2.3 Phenotypic classification

Freezing behaviour, defined as the cessation of all bodily movements except those necessary for respiration (Fanselow, 1980), was used as a measure of the rats' fear response. Freezing was continuously measured manually by experimenters using a custom-written python script. As for the classifications developed by Rau and colleagues (2005), animals were classified based on freezing during the first 8 min of the extinction session. However, rather than adopting the traditional binary criterion whereby animals are classified as ‘Susceptible’ if they show freezing at least two standard deviations above the mean of unstressed controls (Rau et al., 2005), K-means clustering was applied with an initial range of possible cluster numbers (1-10). The optimal number of clusters (K) was determined using the elbow method, which involved plotting the within-cluster sum of squares (WCSS) for different values of K. The elbow point, where the rate of decrease in WCSS slowed, indicated that K = 3 and K = 2 were the most appropriate values for clustering the SEFL and CUS datasets, respectively (**Supplementary Materials 1**). As a result, K-means clustering was performed with K = 3 to classify SEFL subjects into three groups: resilient (M = 8.52%), intermediate (M = 37.93%) and susceptible (M = 74.19%), and with K = 2 to classify animals in the chronic cohort as Non-Depressive-Like (M = 5.60%) or Depressive-Like (M = 39.76%). Whilst we acknowledge that freezing behaviour alone is insufficient to draw parallels to clinical depression, the CUS procedure is a well-validated rodent paradigm which produces a variety of depression-relevant behaviours (Kompagne et al., 2008) including anhedonia (Willner et al., 1992, 1996) and decreased sucrose preference (Monleon et al., 1995; Willner et al., 1987); hence, this terminology was chosen for consistency with existing literature. The number of animals of each sex and shock group meeting each phenotypic criteria is shown in Table 1.

**Table 1:**
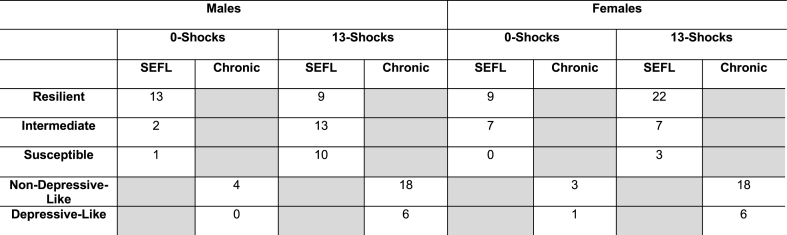
Group numbers for each combination of sex, shocks and phenotype.

### 2.4 Oestrus checking

Prior to each behavioural session, the oestrus stage of female rats was determined through visual inspection of the external genitalia. This is as accurate and reliable as the vaginal smear whilst being quicker and less invasive (Barret et al., 2010). Given the association between menstrual cycle phase and plasma cytokine levels in humans (O'Brien et al., 2007), oestrus stage was also assessed prior to blood sampling to control for any cyclical variations. Analyses revealed no influence of oestradiol levels on any variables of interest. These data are presented in the supplementary materials (**Supplementary Materials 2**).

### 2.5 Blood sampling and analysis

For baseline measurements, blood samples were collected from the lateral tail vein prior to behavioural procedures. Terminal blood samples were obtained immediately after sacrifice. Blood was transferred to 2 ml vacutainer tubes containing heparin (BD Vacutainer™, Fisher Scientific) and centrifuged at 1200 × g for 10 min at 4°C. Resultant plasma was stored at −80°C until processing.

Baseline levels of TNF-α were detected were detected by colorimetric assay (AbCam, ab236712). Terminal levels of the inflammatory cytokines IL-1β, IL-6 and TNF-α, and anti-inflammatory cytokine IL-10, were detected with arigoPLEX® Rat inflammatory cytokine multiplex ELISA kit (arigo Biolaboratories Corp., Hsinchu City, Taiwan) according to manufacturer's instructions. Assays were chosen to match the expected concentration ranges. To accommodate assay throughput limitations, plasma samples were pooled prior to cytokine quantification. Pooling was performed strictly within matched experimental strata defined by shock exposure, cohort, phenotype, and sex to prevent cross-condition mixing and minimise biological variability within pools. For each stratum, samples were assigned to pools sequentially in a randomised order to avoid systematic bias. Small strata containing fewer than the target pool size were retained as singletons or partial pools to preserve group integrity. Larger strata were divided into evenly sized pools (3–4 samples per pool on average) to achieve the required number of assay wells. This approach ensured that each pool was compositionally homogeneous for key experimental factors while maximising the use of available assay capacity. All assays were performed in duplicate. The number of animals contributing to each pool is reported in the supplement (**Supplementary Materials 3)**. Standard curves for each cytokine were generated using the premixed lyophilised standards provided in the kits, and the cytokine concentrations in samples were determined from the appropriate standard curve.

### 2.6 Brain collection and sample preparation

The day after completion of the behavioural procedures, rats were culled by exposure to rising carbon dioxide concentration and exsanguination. Brains were extracted, and flash frozen on dry ice before subsequent storage at −80°c. Brains were sectioned at 150 μm at −20°c within a cryostat (Leica). Samples from the prelimbic cortex (PL), baseolateral amygdala (BLA), infralimbic cortex (IL) and hippocampus (Hpc) were microdissected using a 0.99 mm diameter biopsy punch (Milltex).

### 2.7 Western blotting

Due to insufficient tissue availability for mitochondrial isolation, brains from multiple animals within each experimental group were pooled and processed as crude cell lysates to achieve the recommended total protein concentration (20–50 μg/mL) for downstream analyses. Group sizes ranged from one to 18 animals, and pooled samples consisted of tissue from one to seven animals within the same group. Each pooled or individual sample was treated as a single independent biological replicate for statistical analysis, and all group-level comparisons were performed at the level of these biological units. The number of animals contributing to each pool is reported in **Supplementary Materials 3**. In rare instances, only one biological replicate was available due to the low incidence of specific behavioural classifications following experimental procedures (e.g., 0-shock animals classified as SEFL-susceptible). These cases reflect biological constraints of the classification procedure rather than selective sampling, and this limitation is acknowledged in the interpretation of those comparisons. For completeness, comparisons restricted to groups with minimal n = 3 are presented in **Supplementary Materials 4**.

Microdissected samples were placed into 60 μl of cold lysis buffer supplemented with protease inhibitor cocktail (1:200, AbCam, ab201111). Following sonication, the protein content of each sample was quantified using a spectrophotometer (Nanodrop), and samples with extremely low protein levels were excluded from all subsequent analyses (IL, n = 1: Female, Recent Trauma, 13-Shocks, Susceptible). Before blotting, all samples were diluted to contain the same amount of protein as the most dilute sample (PL = 29.63 μg, IL = 24.06 μg, BLA = 24.38 μg, Hpc = 25.18 μg). Samples were prepared in 2× Laemmli buffer, except for IL samples, which were prepared in 6× Laemmli buffer due to their low protein concentration. This adjustment allowed a greater sample volume to be loaded during western blotting and ensured that all samples were within the required total protein concentration range.

Samples were loaded and separated using a 12% SDS-PAGE and electrotransferred (Bio-Rad) onto a nitrocellulose membrane (Thermo Fisher Scientific). Blots were probed with mouse total OXPHOS antibody cocktail (1:400, Abcam, ab110413) and rabbit anti-TOM70 (1:1000, AbCam, ab289977), diluted in PBS 1% milk which was tested on previously collected samples, with known concentrations of proteins, to ensure that the concentrations of antibodies used were delivering optical density signals that were in the linear range. Signals were visualised using fluorescent anti-mouse (1:20,000, Li-COR IRDye 680RD goat anti-mouse IgG H + L) and anti-rabbit (1:10,000, Li-COR IRDye 800CW goat anti-rabbit IgG H + L) secondary antibodies.

All blots were imaged using the Li-COR Odyssey system. Optical density measurements were made using Li-COR's Image Studio (v5.5.4) for the Odyssey CLx scanner. The quantification of optical densities for the molecular analyses was conducted blind to experimental condition and normalised using TOM-70 expression. All samples were run in duplicate. Analysis of TOM70 expression revealed no significant effects of shock exposure, sex, phenotype, or their interactions (all p > .12), supporting its use as a stable mitochondrial normalisation control in the present study (**Supplementary Materials 5**).

### 2.8 Statistical analyses

All analyses were conducted in R (v2026.01.1 + 403), with the significance level set at α = .05. Given that our aim was to examine biologically relevant variation in behaviour (rather than enforce statistical homogeneity), statistical outliers were not removed from the behavioural dataset.

Preliminary analyses indicated no behavioural differences between animals exposed to remote versus recent trauma. These data are presented in the supplement (**Supplementary Materials 6**). Accordingly, these groups were combined into a single ‘SEFL’ category to reflect the hypothesised similarity and to increase statistical power, while chronic animals were retained as a separate group (Cohort) in all analyses.

Freezing behaviour during conditioning was measured during the 30 s before presentation of the first shock (‘Pre-Shock’), during the 30 s between shocks (‘Post-Shock 1’) and during the 30 s following presentation of the second shock (‘Post-Shock 2’). For the Context B conditioning session, data were analysed using a repeated measures ANOVA, with Time as a within-subject factor and Shocks, Phenotype, Sex and Cohort as between-subjects factors. To compare the increase in freezing between the start and end of fear conditioning, the difference between the 30s prior to the first shock and the 30s following the second shock was calculated, and an independent measures ANOVA conducted to compare these difference scores, with Cohort, Phenotype, Sex and Shocks as between-subjects factors. For the extinction session, freezing was quantified in 10 3-min time bins (Time) and data were analysed using a Gamma GLM with a log-link function, with Time as a within-subject factor and Shocks, Phenotype, Sex and Cohort as between-subjects factors. To examine overall fear reduction during the extinction session, the difference between the first and final minute of extinction was calculated, and an independent measures ANOVA conducted to compare these difference scores, with Cohort, Phenotype, Sex and Shocks as between-subjects factors. Extinction trajectories were analysed using linear mixed-effects models fitted including freezing as the dependent variable and time as a continuous predictor. To capture potential non-linear extinction dynamics, a quadratic term for Time was included. Phenotype and shock condition were included as fixed effects, and all interaction terms with time were modelled to assess differences in extinction trajectories across groups, with subject identity was included as a random intercept to account for repeated measures. Model significance for fixed effects was assessed using Type III analysis of variance with Satterthwaite's approximation for degrees of freedom. Where significant main effects or interactions were observed, post hoc comparisons were conducted using estimated marginal means derived from the fitted model.

To compare recovery of freezing between the end of extinction and the beginning of the retention test, the difference between the final minute of extinction and the first minute of the retention test was calculated, and an independent measures ANOVA conducted to compare these difference scores, with Cohort, Phenotype, Sex and Shocks as between-subjects factors. For the retention test, total freezing was quantified and compared between Phenotype and Shock groups using Gamma GLM with a log link function.

Where ANOVA was used, data were tested for normality using the Shapiro-Wilk test where group sizes allowed, and for homogeneity of variance using Levene's test. If assumptions of normality and homogeneity of variance were only ‘lightly’ violated, ANOVA was used as it is robust to violations of normality and ‘light’ heteroscedasticity (Schmider et al., 2010). In cases where assumptions were strongly violated, GLMs were employed with appropriate distributional families and link functions selected based on the nature and direction of the skew and variance structure. Pairwise comparisons were performed using the Tukey-Kramer test or Holm-Bonferroni method (for repeated measures ANOVAs). Although phenotype and cohort were not fully orthogonal (phenotype categories were present only within specific cohorts), modelling the interaction term (Phenotype * Cohort) allowed us to account for these structured dependencies without assuming independent effects of each factor. This approach ensures that comparisons are restricted to observed phenotype × cohort combinations, avoiding extrapolation to unobserved or structurally missing cells.

Analyses of cytokine concentrations were performed using a four way ANOVA with Sex, Cohort, Phenotype and Shocks as between-subjects factors. Samples with back-calculated concentrations below the lowest standard were reported as < LLOQ and treated as censored in statistical analyses. Influential data points were identified using median absolute deviations (MADs). For each animal, duplicate measurements were first averaged, and these per-animal means were assessed within each Phenotype × Shock group (Phenotype only for baseline measures). Observations exceeding three MADs from the group median were classified as outliers and removed. While behavioural analyses retained all data to capture individual variability, outlier exclusion was applied to ELISA measures due to their known susceptibility to technical error despite duplication. Group-wise MAD was chosen because some phenotype × shock combinations contained very few animals, making approaches such as Cook's D or IQR applied across the entire dataset inappropriate.

For semi-quantitative Western blot analysis, individual optical density readings were normalised to TOM-70 expression for comparison across gels. Optical densities were normalised to the 0-shock, resilient control group (for the SEFL cohorts) and the 0-Shock Non-Depressive-Like control group (for the chronic cohorts) to facilitate interpretation and are expressed as mean (± standard error of the mean (s.e.m.)). Because normalisation was performed relative to cohort-specific controls, protein expression for each cohort was analysed separately using a three-way univariate ANOVA with Sex, Shocks, and Phenotype as factors. As for analysis of plasma data, influential data points were identified based on MADs and observations exceeding three MADs from the group median were excluded from analyses. This ensured that extreme values likely arising from technical variability did not disproportionately influence statistical estimates, while preserving genuine biological variability within groups. Where only a single biological replicate was available for a given group, this value is presented within the constraints of the classification scheme and is not interpreted as a robust estimate of population-level variance.

## 3 Results

### 3.1 Fear conditioning

During contextual fear conditioning, all phenotypic groups exhibited increased freezing following shock administration, indicating successful acquisition of conditioned fear (Phenotype F_(3,134)_ = 3.86, p = .01, η^2^ = 0.07). Freezing was initially low and increased significantly after both shocks in context B (Time: F_(2,248)_ = 136.19, p < .0001, η^2^ = 0.19). Prior exposure to 13 shocks in context A produced higher overall freezing than 0-shock controls (Shock: F_(1,134)_ = 10.41, p = .002, η^2^ = 0.05), an effect maintained throughout conditioning (Post-Shock 1: F_(1,134)_ = 21.34, <0.0001, η^2^ = 0.08; Post-Shock 2: F_(1,134)_ = 4.98, p = .03, η^2^ = 0.02). Notably, this effect was specific to the SEFL cohort (SEFL: F_(1,85)_ = 29.03, p < .0001, η^2^ = 0.10; CUS: F_(1,49)_ = 0.02, p = .89, η^2^ = 0.0001, Fig. 2a and b). Additionally, SEFL animals froze more overall (M = 33.65 ± 2.21%) than CUS animals (M = 20.72 ± 1.92%) regardless of phenotype (Fig. 2c and d; Cohort: F_(1,134)_ = 7.43, p = .007, η^2^ = 0.04), consistent with greater fear sensitisation following punctate trauma. In the SEFL cohort, phenotypic differences were already evident prior to conditioning. Post hoc analyses revealed that while Intermediate and Susceptible groups were initially separable (p.adj = 0.002), they progressively converged over the course of conditioning, such that they were no longer distinguishable at later stages of acquisition (p.adj = 1), whereas Resilient animals diverged from high-freezing phenotypes across the session. In contrast, animals in the CUS cohort displayed broadly comparable levels of conditioned fear acquisition, with a clear separation between phenotypes only observed at the end of conditioning.

**Fig. 1:**
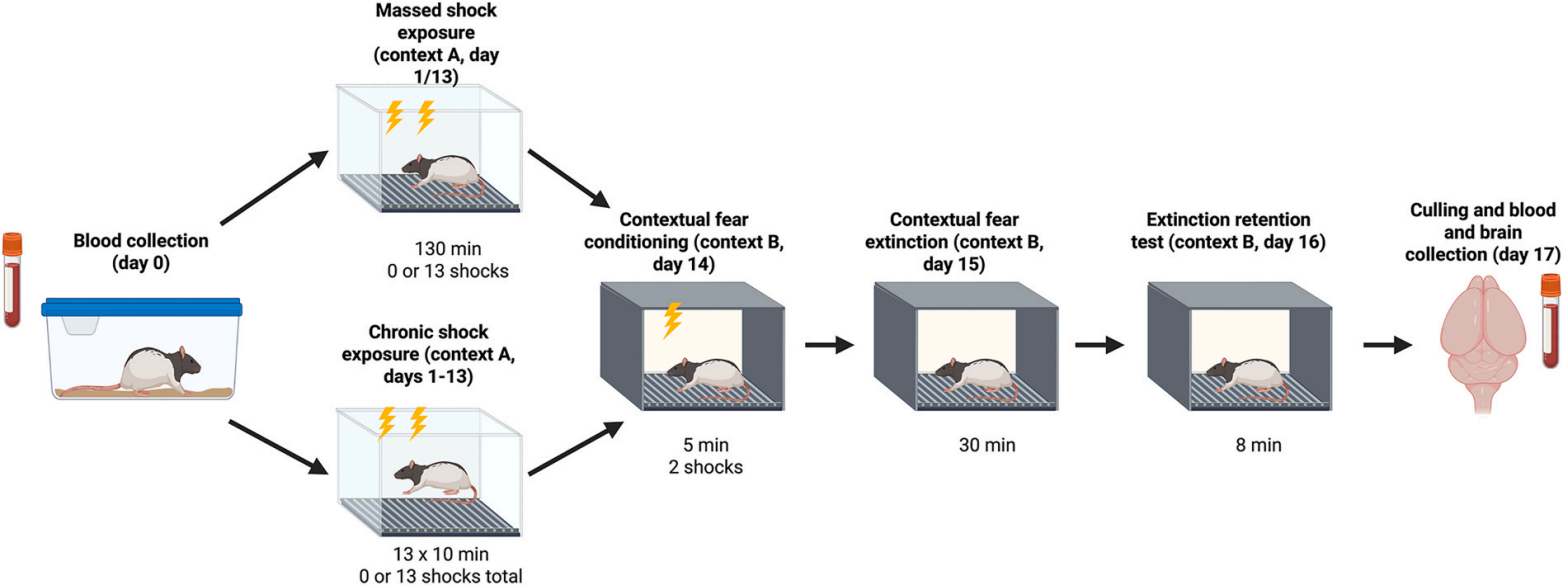
**Behavioural Procedures.** Prior to behavioural procedures, blood samples were collected from the lateral tail vein. Rats assigned to the SEFL groups were exposed to Context A and received 0 or 13 0.5 mA, 0.5s unsignalled footshocks. Rats in the chronic shock groups were exposed to Context A for 10min on 13 consecutive days and received 0, 1 or 2 0.5 mA, 0.5s unsignalled footshocks per session (total number = 0 or 13). From day 14, all cohorts followed the same procedure. On day 14 they were exposed to two 0.5 mA, 0.5s unsignalled footshocks in Context B. 24hrs later, rats were re-exposed to context B for a 30min extinction session. 24hrs later, an extinction retention test was conducted. 24hrs after the retention test, rats were culled by exposure to rising concentration of CO_2_ and exsanguination and brains and blood collected for subsequent analyses. *Created in BioRender. Rye, C.S. (2026).*https://BioRender.com/8r4zqm3.

**Fig. 2:**
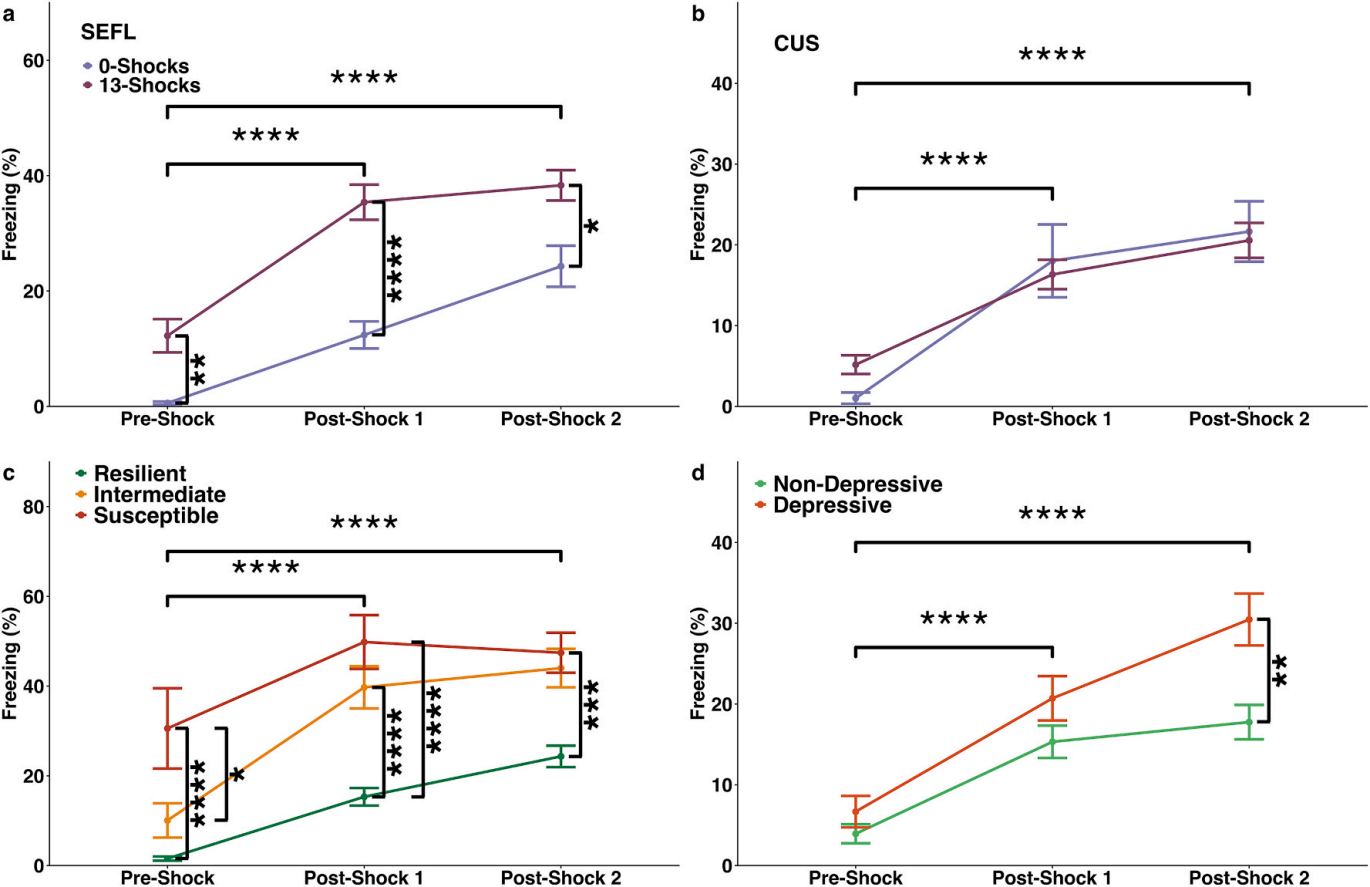
**Fear conditioning is enhanced by prior shock exposure and modulated by phenotype and depressive-like behaviour. a)** In the SEFL cohort, 13-shock animals showed greater freezing than 0-shock controls following both shocks. **b)** In the CUS cohort, freezing increased over time but there were minimal differences between Shock groups. **c)** In the SEFL cohort, freezing was modulated by phenotype, with susceptible animals exhibiting greater freezing than intermediate and resilient groups, and resilient animals showing the lowest levels. **d)** In the CUS cohort, depressive-like animals displayed increased freezing compared to non-depressive-like animals following conditioning, with no differences observed at baseline. Line graphs show data expressed as mean ± s.e.m**.** See figure key for colour mappings used throughout. *p < .05, **p < .01, ***p < .001, ****p < .0001.

A Phenotype × Sex interaction emerged for the increase in freezing (F_(3,134)_ = 3.04, *p* = .03, η^2^ = 0.05), but this did not survive correction. Within the SEFL cohort (Fig. 2c), susceptible animals froze more than resilient animals at all timepoints (all p.adj>0.0009). In the CUS cohort (Fig. 2d), animals classified as Depressive-Like diverged from those classified as Non-Depressive-Like only following the second shock (p.adj = 0.005; Fig. 2d), with no baseline differences. No sex differences (F_(1,134)_ = 0.99, p = .32, η^2^ = 0.006) or robust interactions between sex and phenotype (F_(4,134)_ = 2.33, p = .06, η^2^ = 0.065) were observed.

### 3.2 Fear extinction

**Fig. 3:**
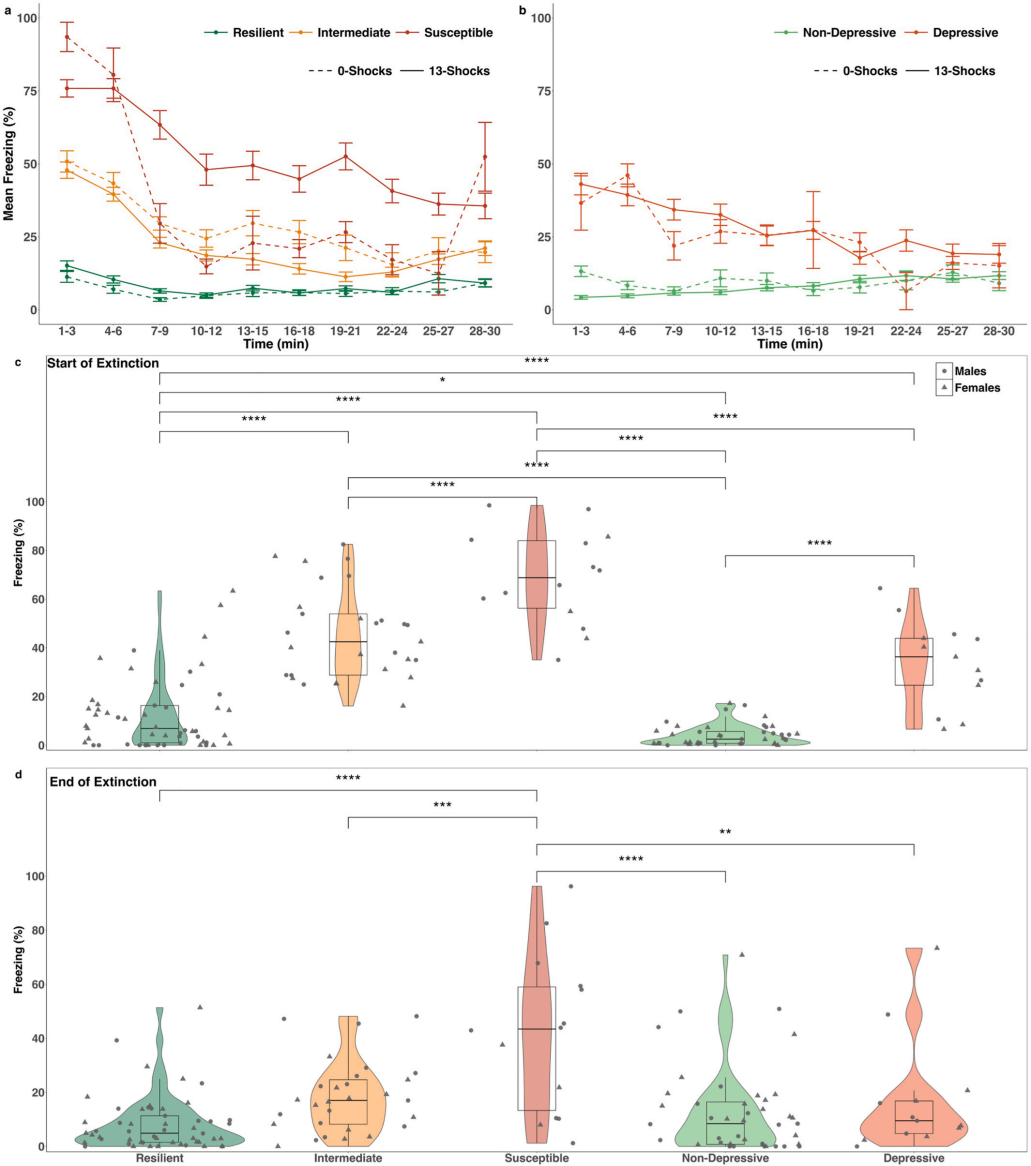
**Freezing during the extinction session differed between phenotypes. a)** Animals classified as susceptible or intermediate showed higher levels of freezing than resilient animals during the extinction session, but no overall difference in freezing was observed between animals exposed to 0-shocks and those exposed to 13-shocks in Context A. **b)** No significant differences in mean freezing were observed between non-depressive-like and depressive-like phenotypes in either the 0-shock or 13-shock groups. **c)** At the start of extinction, differences in freezing were observed between all phenotypes except intermediate and depressive. **d)** By the end of the extinction session, although the overall effect of phenotype remained significant, the difference between the resilient and intermediate groups and non-depressive-like and depressive-like groups was no longer significant, indicating a convergence of their behavioural responses over time. Violin plots show the distribution of the data; box plots indicate the median and interquartile range (IQR), with whiskers extending to 1.5×IQR. Bar plots show data expressed as mean ± s.e.m. Individual data points are overlaid. See figure key for colour and transparency mappings used throughout. *p < .05, **p < .01, ***p < .001, ****p < .0001.

During non-reinforced extinction in Context B, SEFL-susceptible and intermediate animals (Fig. 3a) maintained higher freezing than resilient rats (all *p* < .0006). For the CUS cohort (Fig. 3b), no significant differences in mean freezing were observed between Non-Depressive-Like and Depressive-Like animals (*p* = .21). Comparing across the SEFL and CUS cohorts, Depressive-Like animals did not differ from resilient animals, or intermediate and susceptible groups (*p* = .82). Prior shock exposure had no effect on mean freezing during the session (β = 1.29, SE = 0.78, z = 1.66, p = .098).

Fear reduction across the session (Fig. 3c and d) was associated with phenotype (F_(4,134)_ = 17.4, *p* < .0001, η^2^ = 0.32), with large group differences at the start of extinction (Start: F_(4,134)_ = 85.2, *p* < .0001, η^2^ = 0.68) that narrowed over time (End: F_(4,124)_ = 12.70, *p* < .0001, η^2^ = 0.26). By the end of extinction, no differences could be observed between the freezing of resilient and intermediate groups (p.adj = 0.06, Fig. 3c) or between Non-Depressive-Like and Depressive-Like (p.adj = 0.92; Fig. 3d), indicating a convergence of behavioural response. However, a highly significant pairwise contrast emerged between the intermediate and susceptible groups by the end of extinction (p.adj = 0.0001; Fig. 3d), with animals classified as susceptible exhibiting greater freezing than intermediate animals.

No sex differences were observed in the degree of fear reduction between the start and end of the extinction session (F_(1,134)_ = 0.06, *p* = .81, η^2^ = 0.0003).

Examination of extinction dynamics revealed a significant quadratic effect of time on freezing behaviour (F_(2, 4388)_ = 193.34, p < .0001, ηp^2^ = 0.08), a large main effect of Phenotype (F_(4, 544)_ = 118.91, p < .0001, ηp^2^ = 0.47), and a significant Time^2^ × Phenotype interaction (F_(8, 4388)_ = 63.07, p < .0001, ηp^2^ = 0.10), indicating that extinction trajectories differed across phenotypes in both magnitude and curvature. There was no significant main effect of shock history on extinction dynamics (p = .06), although Shock × Phenotype interactions were present (F_(4, 719)_ = 4.37, p = .002, ηp^2^ = 0.03). Notably, the Time^2^ × Phenotype interaction accounted for a larger proportion of variance than the Shock × Phenotype interaction, suggesting that temporal structure contributed more strongly to between-phenotype differences in extinction than shock history.

Post hoc analyses supported this pattern, revealing robust differences in extinction trajectories across time within phenotypes, whereas effects of shock were more restricted and phenotype dependent. Shock did not significantly influence extinction trajectories in Resilient animals (p.adj = 0.63) or in either CUS cohort (Non-Depressive-Like p.adj = 0.97; Depressive-Like p.adj = 0.72), but did modulate trajectories in Intermediate (p.adj = 0.006) and Susceptible groups (p.adj = 0.0002).

When averaged across shock conditions, Susceptible animals exhibited persistently elevated freezing across extinction relative to all other phenotypes (all p < .0001), reflecting a slower reduction in conditioned responding over time. Intermediate animals showed significantly different extinction trajectories compared with Susceptible (estimate = −29.67, SE = 2.89, t_(177)_ = −10.26, p.adj<0.0001) and Resilient groups (estimate = 11.72, SE = 2.05, t_(177)_ = 5.71, p.adj<0.0001). Similarly, Non-Depressive-Like and Depressive-Like differed in their trajectories (estimate = −17.67, SE = 2.81, t_(177)_ = −6.28, p.adj<0.0001). At the lower end of the distribution, Resilient and Non-Depressive-Like animals did not differ significantly (p = .48), indicating overlapping extinction trajectories characterised by similarly rapid reductions in freezing. In these groups, low and stable freezing likely reflects low initial expression of conditioned fear following acquisition, resulting in limited dynamic range for further within-session reductions rather than absence of extinction learning.

### 3.3 Extinction retention

**Fig. 4:**
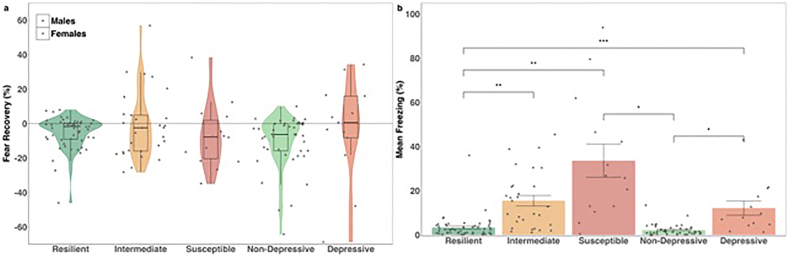
**Test freezing differed between phenotypes despite no differences in fear recovery. a)** All phenotypes recovered fear to the same extent between extinction and testing. Violin plots show the distribution of the data; box plots indicate the median and interquartile range (IQR), with whiskers extending to 1.5×IQR. **b)** At test, differences in freezing behaviour could be observed between behavioural phenotypes. Data expressed as mean ± s.e.m. Individual data points are overlaid. See figure key for colour and transparency mappings used throughout. *p < .05, **p < .01, ***p < .001, ****p < .0001.

Fear recovery between extinction and testing did not differ by phenotype (F_(4,134)_ = 1.60, *p* = .18), shock history (F_(1,134)_ = 0.42, *p* = .52) or sex (F_(1,134)_ = 0.12, *p* = .73; Fig. 4a). However, at test, phenotype predicted fear expression, with intermediate (β = 1.43, SE = 0.72, *z* = 1.98, *p* = .048, 95% CI [0.01, 2.85]), susceptible (β = 2.43, SE = 0.99, *z* = 1.98, *p* = .01, 95% CI [0.48, 4.37]) and Depressive-Like (β = 2.51, SE = 1.21, *z* = 2.08, *p* = .04, 95% CI [0.15, 4.88]) showing higher freezing compared to the resilient reference group (Fig. 4b). There were no effects of shock exposure (β = 0.39, SE = 0.41, *z* = −0.94, *p* = .35, 95% CI [−0.42, 1.20]) or sex (β = −0.02, SE = 0.41, *z* = −0.04, *p* = .97, 95% CI [−0.83, 0.79]). Cohort could not be modelled independently as its variance was fully captured by Phenotype for this outcome.

### 3.4 Plasma cytokines

#### 3.4.1 TNF*-α*

**Fig. 5:**
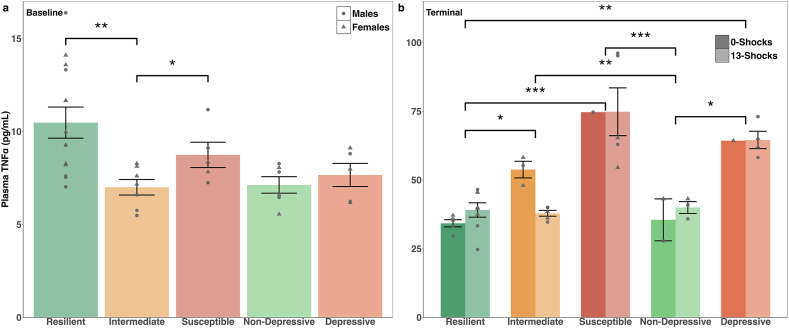
**Graded elevations in circulating TNF-α concentrations (pg/mL) could be seen across behavioural phenotypes at the terminal timepoint. a)** Small differences in circulating TNF- α concentrations were observed at baseline in the SEFL cohort. **b)** Terminal TNF-α concentrations increased progressively from resilient < intermediate < susceptible, and from non-depressive-like < depressive-like, reflecting a continuum of inflammatory activation. Data are expressed as mean ± s.e.m. Individual data points are overlaid. See figure key for colour and transparency mappings used throughout. *p < .05, **p < .01, ***p < .001, ****p < .0001.

Baseline plasma TNF-α concentrations (Fig. 5a) did not differ between males and females (SexFemale: β = 0.51, SE = 0.13, z = 0.41, p = .70, 95% CI [−0.20, 0.30]). However, clear phenotype-related differences emerged: animals later classified as SEFL-Intermediate showed significantly lower baseline TNF-α than both resilient and susceptible groups (resilient > intermediate, p = .003; intermediate < susceptible, p = .03), with no differences between Depressive-Like and Non-Depressive-Like animals or between cohorts (all |β|≤0.0009, p > .99). At terminal sampling (Fig. 5b), this pattern reversed: intermediate and susceptible animals showed higher TNF-α than resilient rats (PhenotypeIntermediate: β = 0.46, SE = 0.18, z = 2.62, p = .02, 95% CI [0.13, 0.82]; PhenotypeSusceptible: β = 0.76, SE = 0.18, z = 4.33, p = .0003, 95% CI [0.43, 1.12]), forming a graded increase from resilient to intermediate to susceptible. Depressive-Like animals also exhibited elevated TNF-α relative to Non-Depressive-Like animals (PhenotypeDepressive-Like: β = 0.98, SE = 0.22, z = 2.02, p = .04, 95% CI [0.56, 1.40]). No effects of sex, shock history, or cohort were observed at endpoint (all |β|≤0.52, p > .20).

#### 3.4.2 IL*-10*

**Fig. 6:**
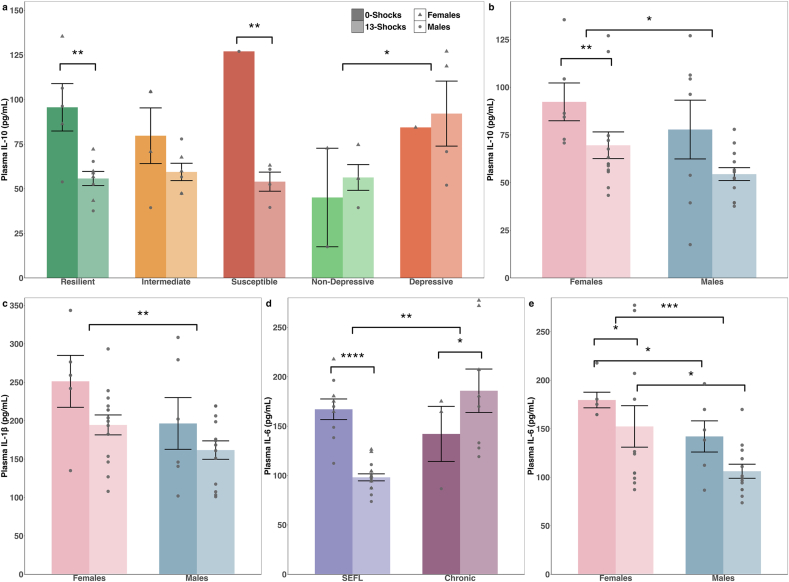
**Terminal cytokine concentrations (pg/mL). a)** Terminal plasma IL-10 levels differed between phenotypes and as a function of shock exposure. **b)** Females showed elevated circulating IL-10 at the terminal sampling point regardless of prior shock exposure**. c)** Females showed elevated circulating IL-1β following behavioural procedures, and this was particularly pronounced in the 0-shock condition. **d)** Prior shock exposure was associated with decreased circulating IL-6 in SEFL animals, but increased IL-6 in animals exposed to chronic stress. **e)** Circulating IL-6 was elevated in females compared to males. Data are expressed as mean ± s.e.m. Individual data points are overlaid. See figure key for colour and transparency mappings used throughout. *p < .05, **p < .01, ***p < .001, ****p < .0001.

Plasma IL-10 at the terminal sampling point (Fig. 6a) showed a phenotype × shock interaction (Phenotype x Shocks: F_(4,21)_ = 4.39, p = .01, η^2^ = 0.22), driven entirely by SEFL animals, with no such interaction observed in rats that had undergone CUS (all p.adj>0.14). Within the SEFL cohort, shock exposure was associated with reduced IL-10 in resilient and susceptible rats (both p.adj = 0.04), whereas chronic stress produced no meaningful change in IL-10 concentration. Terminal IL-10 levels also differed by sex (Fig. 6b; F_(1,21)_ = 5.59, p = .03, η^2^ = 0.07), with females exhibiting higher concentrations than males. No additional phenotype- or shock-related effects were detected.

#### 3.4.3 IL*-1*β

Plasma IL-1β (Fig. 6c) differed by sex and shock history (Sex: F_(1,20)_ = 8.24, p = .009, η^2^ = 0.12; Shocks: F_(1,20)_ = 7.31, p = .01, η^2^ = 0.11). Females showed higher circulating IL-1β than males, and prior shock exposure was associated with reduced IL-1β relative to context exposure alone.

#### 3.4.4 IL*-6*

IL-6 (Fig. 6d) was differentially modulated by shock exposure across stress paradigms (Cohort x Shocks: F_(1,19)_ = 40.49, p < .0001, η^2^ = 0.25). Whilst shock was associated with decreased IL-6 in animals assigned to the SEFL procedure (p.adj<0.0001), this effect was reversed in animals exposed to chronic stress (p.adj = 0.03). A significant interaction between Cohort and Sex (Fig. 6e; F_(1,19)_ = 16.83, p = .0006, η^2^ = 0.10) revealed that sex differences in plasma IL-6 were only observed in the chronic cohort (Chronic: p.adj<0.0001; SEFL: p.adj = 0.29).

### 3.5 Oxidative phosphorylation markers

#### 3.5.1 Prelimbic cortex

**Fig. 7:**
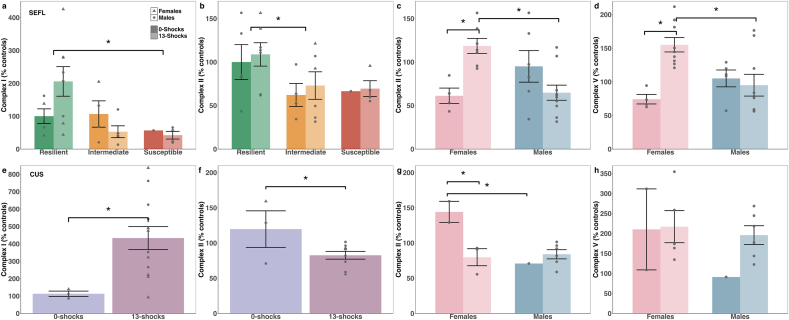
**Expression of OXPHOS subunits in the PL varied between SEFL phenotypes and sexes and as a function of prior shock exposure. (a**–**b)** In animals assigned to the SEFL procedure, phenotypic variations in CI and CII were observed. Susceptible animals had reduced CI expression and intermediate animals lower CII levels than resilient rats. **(c**–**d)** CII and CV expression in SEFL rats was elevated in females compared to males. Effects of shock on CII and CV expression were only observed in females, with prior massed shock associated with greater protein expression in the PL of female rats. **(e**–**f)** Prior chronic shock exposure was associated with elevated CI and decreased CII expression in the PL. **(g**–**h)** CII expression in CUS rats was elevated in females compared to males. Effects of shock on CII expression were only observed in females, with prior massed shock associated with decreased CII expression. No other sex differences were observed in rats assigned to the chronic stress procedure. Data are expressed as mean ± s.e.m. Individual data points are overlaid. See figure key for colour and transparency mappings used throughout. *p < .05, **p < .01, ***p < .001, ****p < .0001.

In the PL of SEFL animals (Fig. 7a and b), mitochondrial protein expression varied as a function of phenotype (Complex I: F_(2,16)_ = 4.04, p = .04, η^2^ = 0.26; Complex II: F_(2,16)_ = 4.63, p = .03, η^2^ = 0.22), with susceptible rats showing reduced Complex I expression relative to resilient animals (p.adj = 0.05), and Complex II expression differing between resilient and intermediate groups (p.adj = 0.03), with no effects of shock (p = .16). In contrast, CUS animals (Fig. 7e and f) showed a distinct pattern: chronic shock increased Complex I expression (F_(1,8)_ = 5.97, p = .04, η^2^ = 0.18), but decreased Complex II expression (F_(1,5)_ = 12.61, p = .02, η^2^ = 0.33), independent of phenotype (all p > .09).

Sex × Shock interactions strongly modulated Complex II expression in both paradigms. In SEFL, females exposed to 13 shocks showed higher CII expression than context-only females (Fig. 7c; p.adj = 0.049), whereas in CUS the same shock manipulation reduced Complex II in females (Fig. 7g; p.adj = 0.02). These interactions were procedure-specific: sex differences emerged only in shocked animals in SEFL (p.adj = 0.01), but only in unshocked animals in CUS (p.adj = 0.04). Complex V expression in SEFL was also sex- and shock-dependent (F_(1,16)_ = 5.79, p = 03, η^2^ = 0.17), with females showing higher CV than males following 13 shocks (Fig. 7d; p.adj = 0.04). In the CUS cohort, no sex differences in CV were observed (Fig. 7h; p = .32).

#### 3.5.2 Basolateral amygdala

**Fig. 8:**
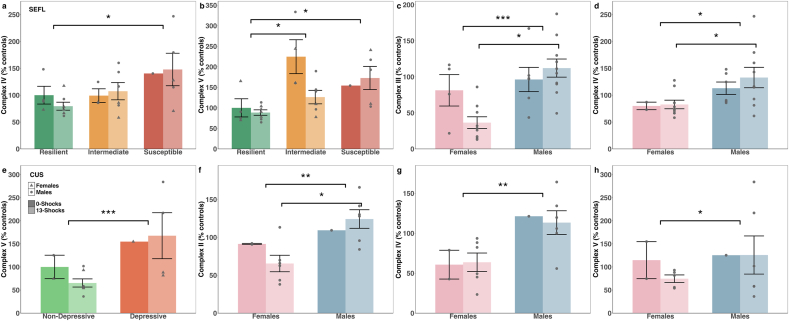
**Expression of OXPHOS subunits in the BLA varied between phenotypes and sexes across stress paradigms. (a**–**b)** In animals assigned to the SEFL procedure, expression of Complex IV (CIV) was elevated in susceptible animals. Intermediate and susceptible rats had heightened Complex V (CV) expression in the BLA compared to resilient rats. **(c**–**d)** Complex III (CIII) and CIV expression in SEFL rats was elevated in males, and sex differences were greater in the 13 shock group. Effects of shock on Complex II (CII) and CV expression were only observed in females, with prior massed shock associated with greater protein expression in the PL of female rats. **(e)** Depressive-like animals showed elevated expression of CV in the BLA compared to their non-depressive-like counterparts. **(f**–**h)** Males assigned to the CUS procedure showed elevated expression of CII, CIV and CV compared to females. Sex differences in CII expression were greater in animals exposed to 13 shocks. Data are expressed as mean ± s.e.m. Individual data points are overlaid. See figure key for colour and transparency mappings used throughout. *p < .05, **p < .01, ***p < .001, ****p < .0001.

In the BLA (Fig. 8), Complex V expression was elevated in both SEFL-susceptible (Fig. 8b) and CUS-Depressive-Like (Fig. 8e) animals (SEFL: (F_(2,16)_ = 6.58, *p* = .008, η^2^ = 0.33; CUS: F_(1,7)_ = 41.3, *p* = .0004, η^2^ = 0.51) compared to resilient and Non-Depressive-Like groups respectively, contrasting with the PL pattern. Complex IV expression (Fig. 8a) also varied by phenotype in the SEFL cohort (F_(2,15)_ = 3.68, *p* = .049, η^2^ = 0.20), with some evidence for higher levels in susceptible rats (p.adj = 0.05). No other phenotype effects were detected (all *p* > .14).

Across both paradigms, OXPHOS expression showed robust sex differences. Males consistently exhibited higher expression of several complexes, with effects most pronounced following 13 shocks. In SEFL animals (Fig. 8c) a significant Shocks × Sex interaction for Complex III expression was observed (F_(1,18)_ = 5.08, *p* = .04, η^2^ = 0.09), indicating that male–female differences were more prominent following trauma exposure (p.adj = 0.0006). This effect was further qualified by a significant Shocks × Sex × Phenotype interaction (F_(1,18)_ = 7.56, *p* = .01, η^2^ = 0.14), with *post hoc* analyses revealing that sex differences were largest among animals exposed to 13 shocks exhibiting an intermediate phenotype (p.adj = 0.04). For Complex IV (Fig. 8d), sex differences were observed (F_(1,15)_ = 9.62, *p* = .007, η^2^ = 0.26), driven by higher Complex IV expression in males relative to females within the 13-shock condition (p.adj = 0.046).

In the CUS cohort, males showed higher Compex II (Fig. 8f; F_(1,8)_ = 14.20, *p* = .006, η^2^ = 0.52), Complex IV (Fig. 8g; F_(1,8)_ = 11.50, *p* = .009, η^2^ = 0.46), and Complex V (Fig. 8h; F_(1,7)_ = 8.08, *p* = .02, η^2^ = 0.10) expression, with Complex V additionally showing a Sex × Phenotype interaction reflecting elevated Complex V in Depressive-Like males (p.adj = 0.0007).

Shock exposure alone did not alter OXPHOS expression when averaged across sex and phenotype. Instead, the effects of shock exposure on OXPHOS expression were contingent on biological factors such as sex and phenotype.

#### 3.5.3 Infralimbic cortex

**Fig. 9:**
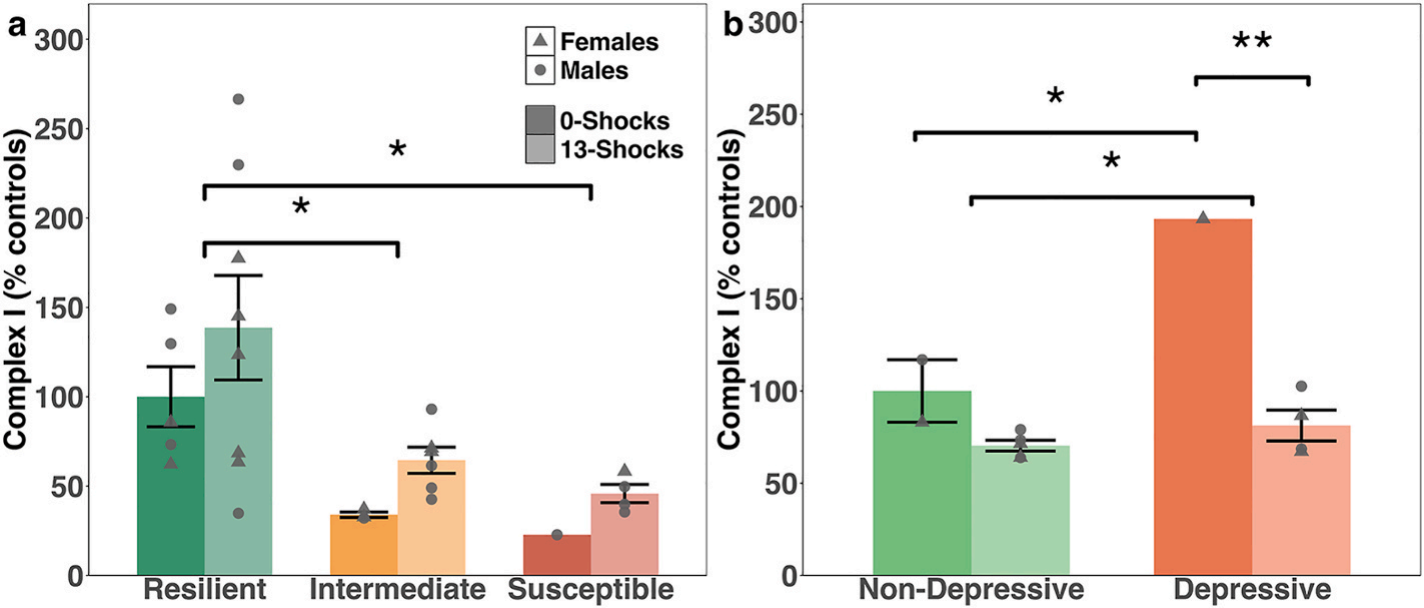
**Expression of CI in the IL varied between phenotypes across stress paradigms. (a)** In animals assigned to the SEFL procedure, expression of Complex I (CI) was decreased in intermediate and susceptible animals. **(b)** Depressive-like animals showed elevated expression of CI in the IL compared to their non-depressive-like counterparts. Data are expressed as mean ± s.e.m. Individual data points are overlaid. See figure key for colour and transparency mappings used throughout. *p < .05, **p < .01, ***p < .001, ****p < .0001.

Phenotypic differences in Complex I expression within the IL (Fig. 9) were evident across both procedures (SEFL: F_(2,16)_ = 7.66, *p* = .005, η^2^ = 0.44; CUS: F_(1,5)_ = 14.6, *p* = .01, η^2^ = 0.48), although the direction of effects differed. Following the SEFL procedure (Fig. 9a), animals classified as intermediate or susceptible exhibited lower Complex I expression than resilient animals (both p.adj = 0.02). In contrast, following the CUS procedure (Fig. 9b), Depressive-Like animals showed increased Complex I expression compared to their Non-Depressive-Like counterparts. This phenotypic difference was especially pronounced in animals not previously exposed to shock in Context A (Phenotype x Shock: F_(1,5)_ = 21.8, *p* = .006, η^2^ = 0.27). Across OXPHOS complexes, no sex differences were observed in protein expression (all *p* > .07).

#### 3.5.4 Hippocampus

**Fig. 10:**
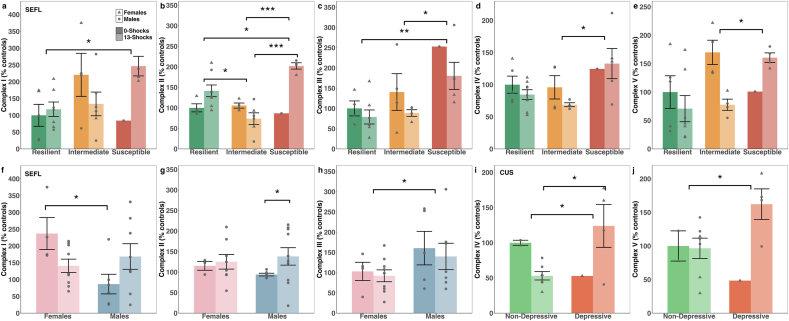
**Broad phenotype-related alterations can be observed in the hippocampus following SEFL, whilst CUS produces more selective mitochondrial changes. (a**–**e)** In animals assigned to the SEFL procedure, expression of CI-CV was elevated in susceptible animals. **(f**–**h)** CI-CIII expression in SEFL rats was modulated by sex and shock exposure. **(i**–**j)** Depressive-like animals showed elevated expression of CIV and CV in the Hpc compared to their non-depressive-like counterparts. Data are expressed as mean ± s.e.m. Individual data points are overlaid. See figure key for colour and transparency mappings used throughout. *p < .05, **p < .01, ***p < .001, ****p < .0001.

In the SEFL cohort, mitochondrial protein expression in the hippocampus (Fig. 10) showed a consistent phenotype-linked increase across multiple complexes. Susceptible animals exhibited higher expression of Complex I, Complex III, Complex IV, and Complex V relative to resilient or intermediate rats (all p.adj<0.04), with Complex II additionally showing a strong phenotype effect (F_(2,16)_ = 12.1, *p* = .0006, η^2^ = 0.39) and a Shock × Phenotype interaction (F_(2,16)_ = 5.56, *p* = .01, η^2^ = 0.18). Several complexes (CI–III) were further modulated by sex and shock exposure (Fig. 10f–h). For Complex I, there was a significant Sex × Shock interaction (F_(1,16)_ = 8.33, *p* = .01, η^2^ = 0.20). Females showed higher Complex I expression than males in the 0-shock condition (p.adj = 0.04), a pattern that was not present in the shock group (p.adj = 0.92), while shock increased CII expression selectively in males (p.adj = 0.04).

In contrast, the CUS model (Fig. 10i and j) produced more selective mitochondrial alterations in the hippocampus. No phenotype, sex, or shock effects were detected for Complexes I–III. Instead, Depressive-Like animals showed elevated Complex IV (F_(1,7)_ = 10.9, *p* = .01, η^2^ = 0.23) and Complex V (F_(1,7)_ = 5.91, *p* = .045, η^2^ = 0.20) expression, with Complex V additionally exhibiting a Sex × Phenotype (F_(1,7)_ = 7.05, *p* = .03, η^2^ = 0.23) interaction driven by higher expression in Depressive-Like females (p.adj = 0.04).

## 4 Discussion

The present study reports the consequences of two experimental interventions in rats to model behavioural, inflammatory and mitochondrial aspects of trauma-related psychopathology. In line with our initial hypothesis, recent and remote trauma exposure produced comparable behavioural outcomes. While this justified pooling the groups for downstream analyses, it suggests that the behavioural consequences of trauma were largely stable across the temporal window examined here. In contrast to our hypothesis however, we observed a lower proportion of susceptible females compared with males within the SEFL paradigm. This finding indicates that vulnerability to stress-enhanced fear learning in this model does not directly recapitulate the higher prevalence of PTSD observed in women. Rather, SEFL susceptibility likely reflects specific components of stress-induced fear sensitisation that may be differentially influenced by sex-dependent neurobiological mechanisms. Nonetheless, across these interventions, stress exposure produced distinct, phenotype-dependent behavioural outcomes, accompanied by alterations in both inflammatory signalling and mitochondrial function. Notably, a tripartite behavioural structure emerged in the SEFL model, capturing meaningful variation in trauma response, whereas chronic stress produced two discrete phenotypes: Non-Depressive-Like and Depressive-Like.

A key finding of the present study is that behavioural phenotypes cannot be fully explained by differences in initial levels of conditioned fear or extinction independently. Whilst behavioural divergence in the SEFL cohort was apparent during the conditioning session, phenotypic differentiation in the CUS cohort emerged more prominently during extinction learning. Furthermore, whilst within the SEFL cohort, Susceptible animals consistently exhibited higher freezing at the start of extinction, our analysis revealed that this alone did not account for the full structure of phenotypic differences. Instead, extinction trajectories were non-linear and significantly modulated by phenotype, suggesting that phenotypic variation reflects not only differences in the expression of conditioned fear at the onset of extinction, but also differences in the temporal dynamics by which fear is updated across the extinction session.

Importantly, interpretation of these effects must also consider conditioning history across stress paradigms. In the CUS condition, lower levels of freezing during extinction relative to SEFL-exposed groups primarily reflect differences in early stress responsivity rather than extinction-specific processes, as CUS-exposed animals did not exhibit the same pattern of fear sensitisation following the second shock during conditioning. Thus, apparent cross-paradigm differences in extinction performance should be interpreted in the context of distinct conditioning-phase dynamics rather than absolute freezing levels alone.

Furthermore, the low levels of conditioned responding observed in Resilient and Non-Depressive-Like animals during extinction occurred in the context of clear fear acquisition during conditioning, indicating that this phenotype reflects adaptive regulation of an acquired fear memory rather than differences in learning. These findings indicate that behavioural phenotypes are best understood as reflecting partially dissociable components of conditioned fear expression, including both initial response magnitude and the temporal structure of extinction.

Furthermore, the observed reductions in IL-10 and IL-6 in PTSD-relevant phenotypes, alongside *increased* IL-6 in depression-relevant states, recapitulate established clinical patterns (Maes et al., 1997; Toft et al., 2022), supporting the translational validity of these models. These findings suggest that individual differences in stress responsivity are shaped by interacting changes in inflammation and cellular energetics, with potential consequences for plasticity and behavioural adaptation.

### 4.1 Stress responses are clustered into distinct behavioural phenotypes

By applying an unsupervised clustering approach to behavioural data, we identified distinct phenotypic structure across models. In the SEFL model, three phenotypes – resilient, intermediate, and susceptible – emerged and captured meaningful biological variation, whereas in the depression model a binary two-cluster solution was observed. The tripartite structure observed following SEFL challenges the traditional binary classification of ‘resilient versus susceptible’ (Rau et al., 2005; Van Assche et al., 2022) and aligns more closely with the dimensional nature of trauma-related symptoms observed in humans (Martalek et al., 2024; Mulder et al., 2013). Rather than assigning individuals into categorical diagnostic groups, our findings suggest that trauma responses exist along a continuum, with a substantial proportion of animals occupying an intermediate, subthreshold state. This intermediate group is particularly informative: behaviourally, they expressed strong fear sensitisation during conditioning, yet showed relatively efficient extinction learning, ultimately reaching low levels of freezing comparable to the resilient phenotype. This pattern is consistent with intact fear memory accompanied by attenuated expression during extinction. In contrast, SEFL-susceptible animals show deficits in extinction learning akin to those experienced by individuals with PTSD (Maren and Holmes, 2016; Milad et al., 2009). This pattern is conceptually consistent with clinical observations that individuals with elevated initial reactivity to trauma may nonetheless show preserved capacity for extinction-based learning processes (Parsons and Ressler, 2013). However, we emphasise that this represents a broad mechanistic parallel rather than a direct translational mapping onto treatment response in patients. The adoption of a three-phenotype framework may therefore be useful for capturing heterogeneity in fear responding within the SEFL paradigm. More broadly, variability in treatment response in PTSD highlights the clinical importance of identifying distinct behavioural and biological subtypes; however, the extent to which the present phenotypes map onto clinical response rates remains speculative.

By contrast, the CUS model did not produce evidence of enhanced fear sensitisation and instead yielded a binary structure based on freezing behaviour. This lack of sensitisation suggests that chronic stress may not amplify learning during a subsequent aversive experience but rather induces a more generalised alteration in behavioural state. The emergence of only two phenotypes further indicates that prolonged stress exposure may reduce behavioural differentiation, overriding more nuanced individual response patterns observed following acute trauma. As such, whereas trauma appears to exaggerate and diversify behavioural responses, chronic stress, within the domain of fear learning and extinction assessed here, may compress these responses into a more constrained profile characterised by the presence or absence of depressive-like features.

### 4.2 Susceptibility to the PTSD-relevant and depressive-like phenotypes may reflect trait-like individual differences

Behaviourally, we replicated core features of the SEFL model, including robust fear sensitisation and extinction impairments in susceptible animals (Perusini et al., 2016; Rau et al., 2005; Rau and Fanselow, 2009; Van Assche et al., 2022). In contrast, chronic stress did not produce fear sensitisation, reinforcing the notion that SEFL and CUS model distinct aspects of trauma-related psychopathology.

Importantly, across models, extinction learning was driven by phenotype rather than the history of uncontrollable stress, supporting the view that vulnerability to stress-induced psychopathology is an intrinsic trait revealed by trauma/stress exposure. This aligns with emerging evidence that differences in neural circuitry (Rye and Milton, 2025), neuropeptide signalling (Lehner et al., 2021), and comorbid psychological traits (Breslau et al., 2013) shape susceptibility to PTSD and depression. Notably, we observed minimal sex differences in overt behaviour, suggesting that while males and females may diverge biologically with respect to inflammatory markers and mitochondrial protein expression, these differences do not necessarily manifest at the level of fear expression following shock administration.

### 4.3 Inflammation may be a potential regulator of stress-related neuroplasticity

Plasma cytokine analyses revealed that inflammation reflects a shared, transdiagnostic mechanism across models, but with phenotype-specific nuances. Baseline TNF-α levels differed across phenotypes, with resilient animals showing the highest concentrations, intermediate animals the lowest, and susceptible animals falling between these extremes, leading us to speculate that inflammatory tone may preconfigure subsequent behavioural trajectories and act as a ‘gate’ on neuroplasticity. Following trauma, SEFL-susceptible animals exhibited the largest TNF-α increase, suggesting heightened inflammatory reactivity. By contrast, the CUS model was characterised by elevated TNF-α in Depressive-Like animals at the terminal sampling point, without baseline differences. This may suggest that inflammatory changes under chronic stress are not pre-existing, but rather develop as a consequence of prolonged stress exposure. This framework aligns with evidence that TNF-α directly modulates structural plasticity; for example, systemic TNF-α administration increases dendritic spine turnover independent of blood–brain barrier disruption (Mauricio Garré et al., 2017). Consequently, SEFL-susceptible animals may possess high plasticity during trauma (facilitating strong fear learning) but reduced plasticity during extinction due to elevated post-trauma inflammation. Intermediate animals may maintain sufficient plasticity across both phases (consistent with enhanced fear learning but intact extinction processes), whereas the tight regulation of inflammatory responses in resilient animals may prevent maladaptive learning and fear sensitisation. The pattern observed in the CUS model is potentially consistent with a shift toward a sustained, low-plasticity state in the depressive-relevant phenotype, rather than the dynamic, phase-specific modulation observed in SEFL. The absence of baseline differences, together with the lack of fear sensitisation, further supports the idea that chronic stress may blunt capacity to engage plasticity from the outset, rather than dysregulating it across learning phases. As such, it is plausible that inflammation in the CUS model may reflect a downstream marker of cumulative stress burden.

Together, these findings suggest that TNF-α may act as a dynamic regulator of plasticity in trauma-related contexts, but as a more sustained constraint under chronic stress, thereby contributing to distinct behavioural and phenotypic outcomes across models. Future work should incorporate temporally resolved cytokine sampling – particularly across conditioning and extinction – to directly test the hypothesis as to whether dynamic inflammatory responses predict behavioural outcomes.

TNF-α is also a key regulator of glutamatergic neurotransmission and adult neurogenesis, playing an essential role in stabilising neural excitability under physiological conditions (Shim et al., 2018; Uzzan and Azab, 2021). However, when elevated, TNF-α may disrupt the balance of excitatory and inhibitory signalling, promoting a shift toward increased activity at AMPA receptors relative to GABA receptors and promoting the expression of Ca^2+^-permeable AMPA receptors (Santello et al., 2011). This shift enhances susceptibility to glutamate excitotoxicity, and is consistent with our previous work demonstrating alterations in glutamate receptor expression in PTSD-relevant phenotypes (Rye and Milton, 2025).

### 4.4 Hippocampal divergence and amygdala convergence define model-specific and shared mitochondrial adaptations to stress

Hippocampal mitochondrial alterations diverged markedly between the SEFL and CUS models, revealing both shared and model-specific patterns of OXPHOS regulation. In the SEFL hippocampus, susceptible animals exhibited a broad upregulation of OXPHOS complexes, particularly I–III. These findings are consistent with, but do not demonstrate, increased energetic demand or compensatory metabolic responses following punctate trauma (Somvanshi et al., 2019). By contrast, hippocampal alterations in the CUS model were more selective, limited to Complexes IV and V and primarily observed in the Depressive-Like phenotype. This pattern suggests that chronic stress may produce specific mitochondrial changes rather than a global shift in respiratory protein expression. Several of these effects were further modulated by sex, indicating that mitochondrial responsiveness is shaped by both biological sex and the nature of the stressor (Amorim et al., 2025).

Despite these differences, both models implicate hippocampal mitochondrial dysfunction as a contributor to stress-related pathology. The shared upregulation of Complexes IV and V across models suggests that alterations in downstream components of oxidative phosphorylation may represent a common vulnerability signature, whereas CI–III alterations may reflect a specific response to acute trauma. One interpretation is that trauma-related phenotypes engage a broader and more rapid mitochondrial response, consistent with the hypothesis that PTSD-relevant states may represent a temporally compressed or ‘accelerated’ form of depressive pathology (Zhang et al., 2022). While our findings do not directly test this relationship, they are compatible with the possibility that this system-wide, rapid-onset, high-amplitude mitochondrial response to trauma could, if unresolved, transition into the more stable but energetically compromised state characteristic of depression, potentially helping to explain the strong comorbidity between depression and PTSD (Rytwinski et al., 2013).

In contrast to the divergence observed in the hippocampus, the BLA exhibited a convergent pattern of mitochondrial adaptation across stress models. Complex V expression was elevated in both SEFL-susceptible and Depressive-Like groups, identifying the BLA as a potential common locus of mitochondrial pathology across the models. This convergence is notable given the central role of the amygdala in mediating affective and fear-related processes (Dalton et al., 2012; Masella et al., 2024; Stevenson, 2011), and aligns with extensive evidence implicating BLA dysfunction in both depressive and trauma-related disorders (Badura-Brack et al., 2018; Bigot et al., 2024; Li et al., 2014; Shin et al., 2006; Yuan et al., 2019).

Mitochondrial alterations in the BLA were strongly modulated by sex, with effects across multiple OXPHOS complexes often amplified by shock exposure. Although behavioural measures did not reveal robust sex differences, these molecular signatures may reflect latent sex-specific vulnerabilities not captured by the behavioural assays employed, consistent with evidence that sex differences in stress-related disorders are often more apparent at the neurobiological level than in overt behaviour (Leuner and Shors, 2013). In the BLA, a robust male-biased expression of multiple OXPHOS complexes was observed across models, particularly in shock-exposed animals. Given the role of the BLA in threat processing and emotional salience (Alexandra Kredlow et al., 2022; Cunningham and Brosch, 2012; Fanselow and LeDoux, 1999; Pelletier et al., 2005), these findings suggest that males may exhibit greater metabolic recruitment of this circuitry under stress, potentially contributing to heightened energetic demand in vulnerable states. This male-biased engagement of amygdala mitochondria is notable considering the higher prevalence of stress-related pathology in females (Tolin and Foa, 2008) and may point to fundamentally different pathways to pathology across sexes. Specifically, vulnerability in males may be characterised by hyperactivation of threat-related circuitry and associated metabolic strain, whereas in females, susceptibility may arise through heightened stress sensitivity, neuroendocrine dysregulation, or dysfunction in regions such as the hippocampus (Irie et al., 2003; Hill et al., 2008). Sex differences in inflammatory signalling may also contribute to the observed mitochondrial effects, as females exhibited heightened cytokine responses, including IL-1β. Such a framework supports the notion that sex differences in stress-related disorders reflect not simply differences in magnitude of response, but divergence in the underlying biological systems through which vulnerability emerges.

Consistent with this interpretation, hippocampal mitochondrial adaptations showed a different pattern of sex dependence. In the CUS model, sex differences were more selective and emerged primarily as a Sex × Phenotype interaction for Complex V, with elevated expression observed in Depressive-Like females. As Complex V reflects the terminal step of oxidative phosphorylation (Das, 2003), its upregulation may indicate a compensatory response to sustained energetic strain rather than enhanced mitochondrial efficiency *per se*.

Together, these findings indicate that mitochondrial adaptations to stress are shaped by both biological sex and regional circuit function. While upstream mitochondrial responses diverge across models and sexes, vulnerability-associated increases in Complex V emerge as a convergent feature across brain regions and stress paradigms, suggesting that changes in the abundance of proteins involved in regulating ATP-producing capacity may represent a common endpoint of stress-related pathology.

Nonetheless, a limitation of the present study is that mitochondrial analyses were restricted to measurements of protein abundance and therefore do not provide direct evidence of altered mitochondrial function. While changes in OXPHOS subunits and related mitochondrial proteins may reflect adaptations in bioenergetic processes, functional measures such as respiratory activity, ATP production, mitochondrial membrane potential, reactive oxygen species generation, or enzymatic activity were not assessed. Consequently, interpretations regarding mitochondrial function should be considered inferential and hypothesis-generating rather than definitive. Future work should aim to directly assess mitochondrial function using complementary approaches such as respirometry and metabolic flux analyses, which will allow a more comprehensive characterisation of the relationship between stress-induced behavioural phenotypes and mitochondrial physiology.

### 4.5 Stress sensitisation is likely mediated by inflammatory and mitochondrial pathways in the prelimbic cortex

A striking regional dissociation emerged within the prefrontal cortex, whereby mitochondrial variation in the infralimbic cortex was limited and largely stress-invariant, while the prelimbic cortex exhibited broad, phenotype-linked alterations that were specific to the SEFL model. Whilst reduced tissue yield and necessary pooling may have limited sensitivity to detect more subtle effects; this pattern identifies the prelimbic cortex as a candidate locus of stress sensitisation rather than a general stress response. This distinction is particularly relevant given that SEFL, unlike CUS, models stress sensitisation – a core feature of PTSD characterised by exaggerated responses to subsequent stressors (Rau et al., 2005; Rau and Fanselow, 2009; Van Assche et al., 2022). Converging with prior evidence of prelimbic glutamatergic dysregulation following SEFL (Rye and Milton, 2025), these findings suggest that mitochondrial adaptations in this region may underpin sensitised fear responding. The prelimbic cortex is well positioned to drive this phenotype, exerting top-down control over fear expression via glutamatergic projections to the basolateral amygdala, thereby amplifying threat-related behaviour (Cheriyan et al., 2016; Milad and Quirk, 2002).

In contrast, the infralimbic cortex displayed minimal phenotype-linked variation, with alterations largely restricted to Complex I expression across stress paradigms. Given that Complex I is a key site of reactive oxygen species generation and is highly sensitive to inflammatory modulation (Fato et al., 2008), this pattern may reflect an early or upstream mitochondrial vulnerability that does not propagate to downstream circuit dysfunction. Indeed, Complex I impairment is known to reduce ATP production while increasing oxidative stress (Kirby et al., 1999), suggesting that infralimbic mitochondria may be affected by stress exposure but remain functionally compensated. This interpretation is supported by the absence of phenotype-specific glutamatergic alterations in this region (Rye and Milton, 2025), indicating that mitochondrial perturbations do not translate into disrupted excitatory signalling. Consistent with this, temporally resolved evidence demonstrates that mitochondrial DNA (mtDNA) alterations – including increased copy number and the accumulation of mutations and deletions – can arise prior to overt cellular dysfunction (West and Shadel, 2017; Westermann, 2008). Together, these findings suggest that mitochondrial changes in the infralimbic cortex reflect an early-stage adaptation that does not reach the threshold required to disrupt excitatory signalling.

We therefore propose a theoretical mechanistic model in which elevated inflammatory tone constrains mitochondrial function in SEFL-susceptible individuals, particularly within the PL, reducing metabolic reserve and impairing calcium buffering capacity. In neurons, which uniquely rely on mitochondrial glutamate oxidation to regulate excitatory load (Divakaruni et al., 2017; Vaglio-Garro et al., 2024), such impairments could theoretically remove a critical metabolic ‘brake’ on glutamate accumulation. Inflammatory signalling, including TNF-α-mediated activation of receptor interacting serine/threonine kinase 1 (RIPK1), may further exacerbate this process, for example by promoting mitochondrial fragmentation via Drp1 (Gong et al., 2024; Lai et al., 2024; Papageorgiou and Filiou, 2024), and thereby reducing calcium buffering capacity and increasing susceptibility to excitotoxic stress(Bravo-Sagua et al., 2017). Within this permissive environment, activity-dependent glutamate release (potentially driven by shock exposure) could preferentially engage excitotoxic pathways.

The selective emergence of these mitochondrial alterations in the PL of SEFL, but not CUS, animals supports this model, indicating that such metabolic vulnerability is specifically associated with stress sensitisation rather than stress exposure *per se*. Whether these alterations represent a pre-existing vulnerability or arise as a consequence of trauma remains unresolved, and longitudinal or baseline assessments will be required to disentangle cause from consequence. However, their phenotype specificity suggests that prelimbic mitochondrial function may serve as a biomarker of susceptibility to PTSD-relevant states. Together, although these findings do not directly test this hypothesis, they support a model in which inflammation-driven mitochondrial dysfunction in the prelimbic cortex interacts with, rather than solely determines, glutamatergic dysregulation, lowering the threshold for excitotoxic signalling via the GluN2B pathway(Vaglio-Garro et al., 2024) and driving persistent stress sensitisation.

### 4.6 A hypothetical framework for pathophysiological mechanisms and therapeutic interventions

We propose that individual differences in mitochondrial reserve capacity and efficiency may pre-exist stress exposure and contribute to vulnerability to stress-related psychopathology. These baseline differences may be partially influenced by variability in inflammatory tone, including circulating TNF-α levels, which are known to interact with mitochondrial function and neural plasticity (Branchi, 2011; Branchi et al., 2024; Vattemi et al., 2013).

Following trauma, SEFL-susceptible individuals may exhibit rapid, circuit-specific mitochondrial adaptations characterised by increased oxidative phosphorylation within the BLA and dysregulated or reduced mitochondrial function within the PL. This pattern is consistent with heightened engagement of threat-processing circuitry alongside reduced top-down regulatory control (Alexandra Kredlow et al., 2022; McLaughlin et al., 2014) and may contribute to elevated inflammatory signalling and oxidative stress. In turn, inflammation–mitochondrial interactions may exacerbate cellular stress, impair mitochondrial integrity, and disrupt calcium buffering, leading to altered synaptic plasticity and impaired encoding of fear and safety learning (Miller et al., 2018; Miller and Sadeh, 2014; Raffaele et al., 2020; Santello et al., 2011).

In contrast, chronic stress and depression-relevant phenotypes appear to be associated with more selective and progressive mitochondrial alterations, consistent with a more constrained and potentially compensatory energetic response. This pattern suggests that depressive-like states may emerge through cumulative energetic inefficiency rather than the broad system-wide mitochondrial activation observed following acute trauma. Such gradual bioenergetic dysregulation may contribute to reduced metabolic flexibility, impaired synaptic plasticity, and the progressive emergence of affective and cognitive deficits characteristic of chronic stress exposure.

From a therapeutic perspective, these findings suggest that interventions targeting inflammatory signalling, including TNF-α modulation, as well as mitochondria-targeted antioxidants, may hold transdiagnostic potential across stress-related disorders. However, the present data also indicate that PTSD- and depression-relevant phenotypes arise through partially distinct circuit-level adaptations, with acute trauma preferentially engaging amygdala–prefrontal systems and chronic stress producing more selective hippocampal mitochondrial alterations. Consequently, while upstream inflammatory and mitochondrial pathways may represent shared therapeutic targets, their efficacy is likely to depend on the underlying circuit architecture and temporal dynamics of pathology.

**Fig. 11:**
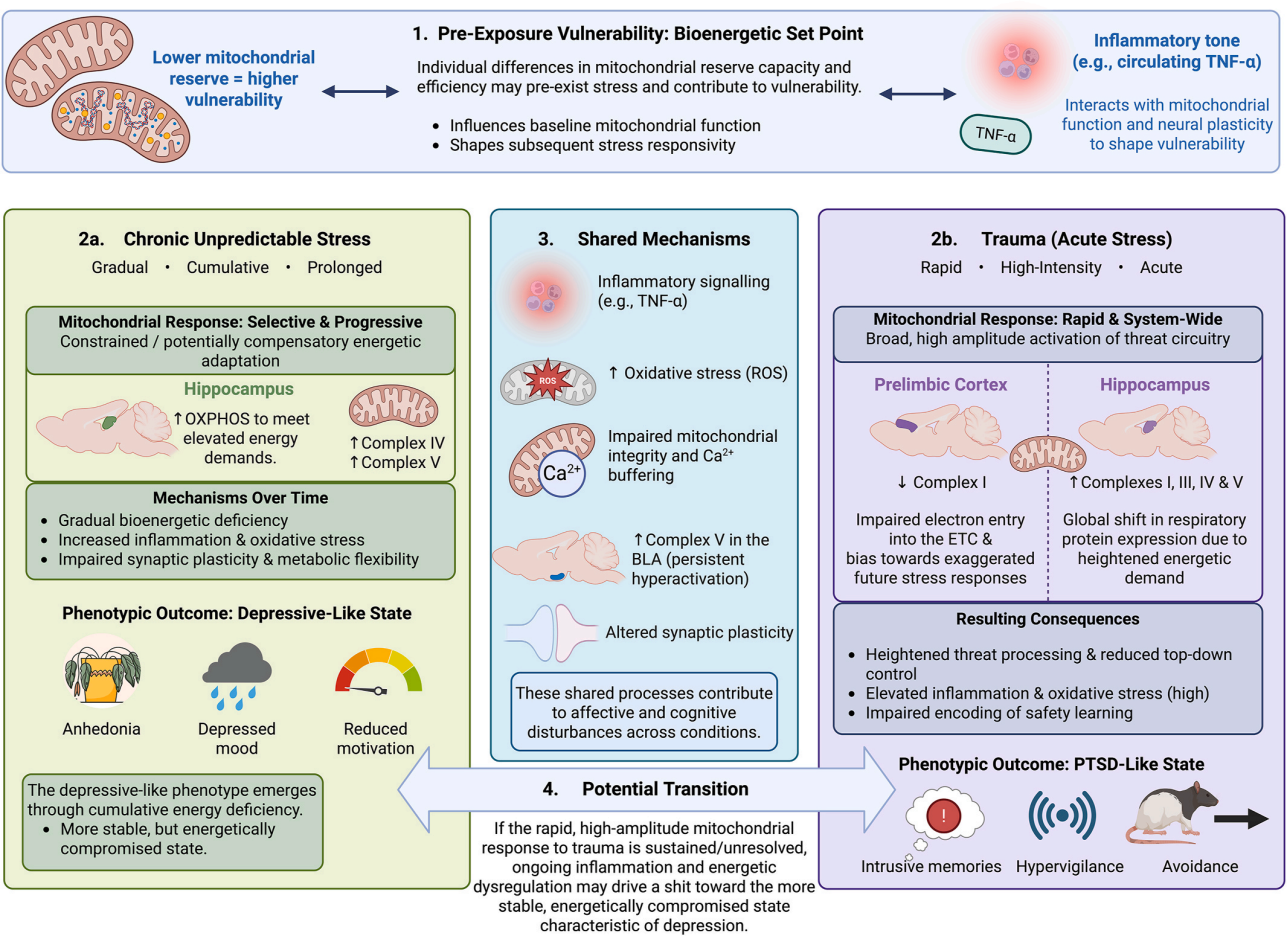
**Hypothesised divergent and convergent mitochondrial pathways to PTSD- and depression-like phenotypes following trauma and chronic stress.** Schematic illustrating how pre-existing differences in mitochondrial reserve capacity and inflammatory tone shape vulnerability to stress-related psychopathology. Acute trauma induces a rapid, high-amplitude, circuit-specific mitochondrial response, leading to PTSD-like phenotypes, whereas chronic unpredictable stress produces gradual, selective mitochondrial dysfunction associated with depression-like states. Despite these distinct pathways, both converge on shared inflammatory and bioenergetic mechanisms. Sustained dysregulation following trauma may drive a transition from PTSD-to depression-like phenotypes, providing a potential basis for their comorbidity. *NB:* The model presented here is intended as a conceptual and hypothesis-generating framework rather than a direct mechanistic interpretation of the current experimental data. It integrates the present findings with existing literature to highlight potential pathways and therapeutic avenues for future investigation. Created in BioRender. Rye, C.S. (2026). https://BioRender.com/71xtm46.

To integrate the present findings with existing literature, we present a conceptual and hypothesis-generating model that outlines potential mechanisms and therapeutic implications (Fig. 11). This framework is not intended to represent a direct mechanistic inference from the current dataset, but rather to highlight directions for future empirical testing.

## 5 Conclusion

A key advance of this work is the use of a clustering-based approach to define stress-responsive behavioural phenotypes in a data-driven manner, moving beyond arbitrary binary thresholds of vulnerability. This dimensional framework revealed distinct behavioural profiles, particularly in SEFL, that were not apparent using standard approaches, highlighting the heterogeneous nature of stress-related behaviour. While a limitation of the current study was the restriction of behavioural readouts to freezing behaviour, limiting our ability to distinguish between adaptive defensive responses to stress-induced pathology, other studies have incorporated additional behavioural assays to capture broader aspects of defensive responding (see for example, Dirven et al., 2022; Dutcher et al., 2023); the present focus therefore prioritised experimental precision and comparability across conditions. Importantly, these phenotypes mapped onto distinct patterns of mitochondrial and inflammatory signalling, indicating that behavioural variability both within and across models reflects underlying biological state differences. Within this framework, inflammatory signalling may regulate neuroplasticity across multiple stress-related phenotypes, shaping how stress exposure is encoded across distributed neural circuits and contributing to the emergence of distinct behavioural states. Importantly, stress sensitisation following SEFL appears to be mediated by integrated inflammatory and mitochondrial pathways within the prelimbic cortex. Consequently, PL may represent a key circuit-level target for interventions aimed at restoring adaptive control over limbic responses in PTSD.

These findings support a multi-level model in which behavioural phenotypes identified through clustering reflect underlying inflammatory–mitochondrial states distributed across the hippocampus, amygdala, and prelimbic cortex. This framework positions mitochondrial bioenergetics and immune signalling as key mechanisms through which stress exposure is translated into distinct yet overlapping trajectories of vulnerability and resilience.

## CRediT authorship contribution statement

**Charlotte S. Rye:** Conceptualization, Data curation, Formal analysis, Investigation, Methodology, Software, Visualization, Writing – original draft, Writing – review & editing. **Laetitia H.E. Ward:** Investigation, Writing – review & editing. **Clara Velázquez-Sánchez:** Investigation, Writing – review & editing. **Jeffrey W. Dalley:** Methodology, Supervision, Writing – review & editing. **Amy L. Milton:** Conceptualization, Funding acquisition, Methodology, Supervision, Writing – review & editing.

## Funding

This work was supported by the UK Research and Innovation's Biological and Biotechnology Research Council under grant number BB/W001195/1.

## Declaration of competing interest

The authors declare no conflicting interests.

## Data Availability

All data accompanying this publication are available at the University of Cambridge data repository (http://doi.org/10.17863/CAM.129792).

## References

[bib1] Alexandra Kredlow M., Fenster R.J., Laurent E.S., Ressler K.J., Phelps E.A. (2022). Prefrontal cortex, amygdala, and threat processing: implications for PTSD. Neuropsychopharmacology.

[bib2] Amorim F.E., Rye C.S., Milton A.L. (2025). Mitochondrial dysfunction in PTSD: a mechanism to understand trauma susceptibility?. Psychopharmacology.

[bib3] Badura-Brack A., McDermott T.J., Heinrichs-Graham E., Ryan T.J., Khanna M.M., Pine D.S. (2018). Veterans with PTSD demonstrate amygdala hyperactivity while viewing threatening faces: a MEG study. Biological Psycholoy.

[bib4] Bangasser D.A., Valentino R.J. (2014). Sex differences in stress-related psychiatric disorders: neurobiological perspectives. Front. Neuroendocrinol..

[bib5] Barret K.E., Barman S.M., Boitano S., Brooks H.L. (2010). *Ganong's Review of Medical Physiology* (24Th Editi).

[bib6] Bauer M.E., Teixeira A.L. (2019). Inflammation in psychiatric disorders: what comes first?. Ann. N. Y. Acad. Sci..

[bib7] Bersani F.S., Morley C., Lindqvist D., Epel E.S., Picard M., Yehuda R., Flory J., Bierer L.M., Makotkine I., Abu-Amara D., Coy M., Reus V.I., Lin J., Blackburn E.H., Marmar C., Wolkowitz O.M., Mellon S.H. (2016). Mitochondrial DNA copy number is reduced in Male combat veterans with PTSD. Prog. Neuro Psychopharmacol. Biol. Psychiatr..

[bib8] Bigot M., De Badts C.H., Benchetrit A., Vicq É., Moigneu C., Meyrel M., Wagner S., Hennrich A.A., Houenou J., Lledo P.M., Henry C. (2024). Disrupted basolateral amygdala circuits supports negative valence bias in depressive states. Transl. Psychiatry.

[bib9] Branchi I. (2011). The double edged sword of neural plasticity: increasing serotonin levels leads to both greater vulnerability to depression and improved capacity to recover. Psychoneuroendocrinology.

[bib10] Branchi I., Viglione A., Vai B., Cirulli F., Benedetti F., Poggini S. (2024). Breaking free from the inflammatory trap of depression: regulating the interplay between immune activation and plasticity to foster mental health. Neuroscience Applied.

[bib11] Bravo‐Sagua R., Parra V., López‐Crisosto C., Díaz P., Quest A.F., Lavandero S. (2017). Calcium transport and signaling in mitochondria. Compr. Physiol..

[bib12] Breslau N., Troost J.P., Bohnert K., Luo Z. (2013). Influence of predispositions on post-traumatic stress disorder: does it vary by trauma severity?. Psychol. Med..

[bib13] Cardinal R.N., Aitken M.R.F. (2010). Whisker: a client-server high-performance multimedia research control system. Behav. Res. Methods.

[bib14] Cheriyan J., Kaushik M.K., Ferreira A.N., Sheets P.L. (2016). Specific targeting of the basolateral amygdala to projectionally defined pyramidal neurons in prelimbic and infralimbic cortex. eNeuro.

[bib15] Cigna (2019). Chronic stress: are we reaching health system burnout. https://www.cignaglobal.com/static/docs/pdf/cigna-asia-care-report-18-nov.pdf?WT.z_nav=news-room%252Fpress-releases%252F2020%252Fstress-related-illness-the-biggest-health-expenditure-in-the-uk-annually.html%253BBody%253BChronic%2520Stress%253A%2520Are%252.

[bib16] Cunningham W.A., Brosch T. (2012). Motivational salience: Amygdala tuning from traits, needs, values, and goals. Curr. Dir. Psychol. Sci..

[bib17] Dalton G.L., Wu D.C., Wang Y.T., Floresco S.B., Phillips A.G. (2012). NMDA GluN2A and GluN2B receptors play separate roles in the induction of LTP and LTD in the amygdala and in the acquisition and extinction of conditioned fear. Neuropharmacology.

[bib18] Das A.M. (2003). Regulation of the mitochondrial ATP-Synthase in health and disease. Mol. Genet. Metabol..

[bib19] Dirven B.C.J., Botan A., van der Geugten D., Kraakman B., van Melis L., Merjenburgh S., van Rijn R., Waajen L., Homberg J.R., Kozicz T., Henckens M.J.A.G. (2022). Longitudinal assessment of amygdala activity in mice susceptible to trauma. Psychoneuroendocrinology.

[bib20] Divakaruni A.S., Wallace M., Buren C., Martyniuk K., Andreyev A.Y., Li E., Fields J.A., Cordes T., Reynolds I.J., Bloodgood B.L., Raymond L.A. (2017). Inhibition of the mitochondrial pyruvate carrier protects from excitotoxic neuronal death. JCB (J. Cell Biol.).

[bib21] Dmytriv T.R., Tsiumpala S.A., Semchyshyn H.M., Storey K.B., Lushchak V.I. (2023). Mitochondrial dysfunction as a possible trigger of neuroinflammation at post-traumatic stress disorder (PTSD). Front. Physiol..

[bib22] Dutcher E.G., Lopez-Cruz L., Pama E.A.C., Lynall M.E., Bevers I.C.R., Jones J.A., Khan S., Sawiak S.J., Milton A.L., Clatworthy M.R., Robbins T.W., Bullmore E.T., Dalley J.W. (2023). Early-life stress biases responding to negative feedback and increases amygdala volume and vulnerability to later-life stress. Transl. Psychiatry.

[bib23] Fanselow M.S. (1980). Conditional and unconditional components of post- shock freezing. Pavlovian J. Biol. Sci..

[bib24] Fanselow M.S., LeDoux J.E. (1999). Why we think plasticity underlying Pavlovian fear conditioning occurs in the basolateral amygdala. Neuron.

[bib25] Fato R., Bergamini C., Leoni S., Strocchi P., Lenaz G. (2008). Generation of reactive oxygen species by mitochondrial complex I: implications in neurodegeneration. Neurochem. Res..

[bib26] Freed M.C., Goldberg R.K., Gore K.L., Engel C.C., Preedy V.R., Watson R.R. (2010). Handbook of Disease Burdens and Quality of Life Measures.

[bib27] Garcia N.M., Walker R.S., Zoellner L.A. (2018). Estrogen, progesterone, and the menstrual cycle: a systematic review of fear learning, intrusive memories, and PTSD. Clin. Psychol. Rev..

[bib28] Gong Q., Ali T., Hu Y., Gao R., Mou S., Luo Y., Yang C., Li A., Li T., Hao L.L., He L., Yu X., Li S. (2024). RIPK1 inhibition mitigates neuroinflammation and rescues depressive-like behaviors in a mouse model of LPS-Induced depression. Cell Commun. Signal..

[bib29] Hill M.N., Miller G.E., Ho W.S., Gorzalka B.B., Hillard C.J. (2008). Serum endocannabinoid content is altered in females with depressive disorders: a preliminary report. Pharmacopsychiatry.

[bib30] Irie M., Asami S., Ikeda M., Kasai H. (2003). Depressive state relates to female oxidative DNA damage via neutrophil activation. Biochem. Biophys. Res. Commun..

[bib31] Kawai T., Akira S. (2007). Signaling to NF-kappaB by toll-like receptors. Trends Mol. Med..

[bib32] Kirby D., Crawford M., Cleary M.A., Dahl H.H., Dennett X., Thorburn D.R. (1999). Respiratory chain complex I deficiency: an underdiagnosed energy generation disorder. Neurology.

[bib33] Kompagne H., Bardos G., Szenasi G., Gacsalyi I., Harsing L.G., Levay G. (2008). Chronic mild stress generates clear depressive but ambiguous anxiety-like behaviour in rats. Behav. Brain Res..

[bib34] Lai K., Wang J., Lin S., Chen Z., Lin G., Ye K., Yuan Y., Lin Y., Zhong C.Q., Wu J., Ma H. (2024). Sensing of mitochondrial DNA by ZBP1 promotes RIPK3-mediated necroptosis and ferroptosis in response to diquat poisoning. Cell Death Differ..

[bib35] Lehner M., Skórzewska A., Wisłowska-Stanek A. (2021). Sex-related predisposition to post-traumatic stress disorder Development—The role of neuropeptides. Int. J. Environ. Res. Publ. Health.

[bib36] Leuner B., Shors T.J. (2013). Stress, anxiety, and dendritic spines: what are the connections?. Neuroscience.

[bib37] Li H., Li X., Smerin S.E., Zhang L., Jia M., Xing G., Su Y.A., Wen J., Benedek D., Ursano R. (2014). Mitochondrial gene expression profiles and metabolic pathways in the amygdala associated with exaggerated fear in an animal model of PTSD. Front. Neurol..

[bib38] Li P., Zhao Y., Wu X., Xia M., Fang M., Iwasaki Y., Sha J., Chen Q., Xu Y., Shen A. (2012). Interferon gamma (IFN-γ) disrupts energy expenditure and metabolic homeostasis by suppressing SIRT1 transcription. Nucleic Acids Res..

[bib39] Lushchak O., Strilbytska O., Koliada A., Storey K.B. (2023). An orchestrating role of mitochondria in the origin and development of post-traumatic stress disorder. Front. Physiol..

[bib40] Maes M., Bosmans E., De Jongh R., Kenis G., Vandoolaeghe E., Neels H. (1997). Increased serum IL-6 and IL-1 receptor antagonist concentrations in major depression and treatment resistant depression. Cytokine.

[bib41] Maes M., Lambrechts J., Bosmans E., Jacobs J., Suy E., Vandervorst C., Dejonckheere C., Minner B., Raus J. (1991).

[bib42] Maren S., Holmes A. (2016). Stress and fear extinction. Neuropsychopharmacology.

[bib43] Martalek A., Dubertret C., Fovet T., Le Strat Y., Tebeka S. (2024). Distressing memories: a continuum from wellness to PTSD. J. Affect. Disord..

[bib44] Masella G., Silva F., Corti E., Azkona G., Madeira M.F., Tomé Â.R., Ferreira S.G., Cunha R.A., Duarte C.B., Santos M. (2024). The amygdala NT3-TrkC pathway underlies inter-individual differences in fear extinction and related synaptic plasticity. Mol. Psychiatr..

[bib45] Mauricio Garré J., Silva H.M., Lafaille J.J., Yang G. (2017). CX3CR1+ monocytes modulate learning and learning-dependent dendritic spine remodeling via TNF-α. Nat. Med..

[bib46] McLaughlin K.A., Busso D.S., Duys A., Green J.G., Alves S., Way M., Sheridan M.A. (2014). Amygdala response to negative stimuli predicts ptsd symptom onset following a terrorist attack. Depress. Anxiety.

[bib47] Milad M.R., Pitman R.K., Ellis C.B., Gold A.L., Shin L.M., Lasko N.B., Zeidan M.A., Handwerger K., Orr S.P., Rauch S.L. (2009). Neurobiological basis of failure to recall extinction memory in posttraumatic stress disorder. Biol. Psychiatry.

[bib48] Milad M.R., Quirk G.J. (2002). Neurons in medial prefrontal cortex signal memory for fear extinction. Nature.

[bib49] Miller A.H. (2020). Beyond depression: the expanding role of inflammation in psychiatric disorders. World Psychiatry.

[bib50] Miller M.W., Lin A.P., Wolf E.J., Miller D.R. (2018). Oxidative stress, inflammation, and neuroprogression in chronic PTSD. Harv. Rev. Psychiatr..

[bib51] Miller M.W., Sadeh N. (2014). Traumatic stress, oxidative stress and post-traumatic stress disorder: neurodegeneration and the accelerated-aging hypothesis. Mol. Psychiatr..

[bib52] Monleon S., D'Aquila P., Parra A., Simon V.M., Brain P.F., Willner P. (1995). Attenuation of sucrose consumption in mice by chronic mild stress and its restoration by imipramine. Psychopharmacology (Berl).

[bib53] Moring J.C., Nason E., Hale W.J., Wachen J.S., Dondanville K.A., Straud C., Moore B.A., Mintz J., Litz B.T., Yarvis J.S., Young-McCaughan S. (2019). Conceptualizing comorbid PTSD and depression among treatment-seeking, active duty military service members. J. Affect. Disord..

[bib54] Mulder R., Fergusson D., Horwood J. (2013). Post-traumatic stress disorder symptoms form a traumatic and non-traumatic stress response dimension. Aust. N. Z. J. Psychiatr..

[bib55] O'Brien S.M., Fitzgerald P., Scully P., Landers A., Scott L.V., Dinan T.G. (2007). Impact of gender and menstrual cycle phase on plasma cytokine concentrations. Neuroimmunomodulation.

[bib56] Papageorgiou M.P., Filiou M.D. (2024). Mitochondrial dynamics and psychiatric disorders: the missing link. Neurosci. Biobehav. Rev..

[bib57] Parsons R.G., Ressler K.J. (2013). Implications of memory modulation for post-traumatic stress and fear disorders. Nat. Neurosci..

[bib58] Pelletier J.G., Likhtik E., Filali M., Pare D. (2005). Lasting increases in basolateral amygdala activity after emotional arousal: implications for facilitated consolidation of emotional memories. Learn. Mem..

[bib59] Peng T.I., Jou M.J., Sheu S.S., Greenamyre J.T. (1998). Visualization of NMDA receptor-induced mitochondrial calcium accumulation in striatal neurons. Exp. Neurol..

[bib60] Perusini J.N., Meyer E.M., Long V.A., Rau V., Nocera N., Avershal J., Maksymetz J., Spigelman I., Fanselow M.S. (2016). Induction and expression of fear sensitization caused by acute traumatic stress. Neuropsychopharmacology.

[bib61] Raffaele S., Lombardi M., Verderio C., Fumagalli M. (2020). TNF production and release from microglia via extracellular vesicles: impact on brain functions. Cells.

[bib62] Raison C.L., Capuron L., Miller A.H. (2006). Cytokines sing the blues: inflammation and the pathogenesis of depression. Trends Immunol..

[bib63] Rau V., DeCola J.P., Fanselow M.S. (2005). Stress-induced enhancement of fear learning: an animal model of posttraumatic stress disorder. Neurosci. Biobehav. Rev..

[bib64] Rau V., Fanselow M.S. (2009). Exposure to a stressor produces a long lasting enhancement of fear learning in rats. Stress.

[bib65] Rye C.S., Milton A.L. (2025). Glutamate receptor expression in the PL-BLA circuit is associated with susceptibility to showing the PTSD-Relevant phenotype. Neurobiol. Learn. Mem..

[bib66] Rytwinski N.K., Scur M.D., Feeny N.C., Youngstrom E.A. (2013). The co‐occurrence of major depressive disorder among individuals with posttraumatic stress disorder: a meta‐analysis. J. Trauma Stress.

[bib67] Santello M., Bezzi P., Volterra A. (2011). TNFα controls glutamatergic gliotransmission in the hippocampal dentate gyrus. Neuron.

[bib68] Schmider E., Ziegler M., Danay E., Beyer L., Buhner M. (2010). Is it really robust? Reinvestigating the robustness of ANOVA against violations of the normal distribution assumption. Methodology.

[bib69] Shim H.G., Jang S.S., Kim S.H., Hwang E.M., Min J.O., Kim H.Y., Kim Y.S., Ryu C., Chung G., Kim Y.S., Yoon B.E., Kim S.J. (2018). TNF-α increases the intrinsic excitability of cerebellar purkinje cells through elevating glutamate release in bergmann glia. Sci. Rep..

[bib70] Shin L.M., Rauch S.L., Pitman R.K. (2006). Amygdala, medial prefrontal cortex, and hippocampal function in PTSD. Ann. N. Y. Acad. Sci..

[bib71] Somvanshi P.R., Mellon S.H., Flory J.D., Abu-Amara D., Wolkowitz O.M., Yehuda R., Jett M., Hood L., Marmar C., Doyle F.J. (2019). Mechanistic inferences on metabolic dysfunction in posttraumatic stress disorder from an integrated model and multiomic analysis: role of glucocorticoid receptor sensitivity. American Journal of Physiology - Endocrinology and Metabolism.

[bib72] Stander V.A., Thomsen C.J., Highfill-McRoy R.M. (2014). Etiology of depression comorbidity in combat-related PTSD: a review of the literature. Clin. Psychol. Rev..

[bib73] Stevenson C.W. (2011). Role of amygdala-prefrontal cortex circuitry in regulating the expression of contextual fear memory. Neurobiol. Learn. Mem..

[bib74] Toft H., Bramness J.G., Lien L. (2022). Levels of peripheral circulating IL-6 and IL-10 decrease over time despite high depression burden in PTSD patients. Neuropsychiatric Dis. Treat..

[bib75] Tolin D.F., Foa E.B. (2008). Sex differences in trauma and posttraumatic stress disorder: a quantitative review of 25 years of research. Psychol. Trauma, Theory Res. Pract. Pol..

[bib76] Uzzan S., Azab A.N. (2021). Anti-tnf-α compounds as a treatment for depression. Molecules.

[bib77] Vaglio-Garro A., Kozlov A.V., Smirnova Y.D., Weidinger A. (2024). Pathological interplay between inflammation and mitochondria aggravates glutamate toxicity. Int. J. Mol. Sci..

[bib78] Van Assche I.A., Padilla M.S., Stupart O.S., Milton A.L. (2022). Refinement of the stress-enhanced fear learning model of post-traumatic stress disorder: a behavioral and molecular analysis. Lab. Anim..

[bib79] Vattemi G., Marini M., Ferreri N.R., Hao S., Malatesta M., Meneguzzi A., Guglielmi V., Fava C., Minuz P., Tomelleri G. (2013). Overexpression of TNF-α in mitochondrial diseases caused by mutations in mtDNA: evidence for signaling through its receptors on mitochondria. Free Radic. Biol. Med..

[bib80] West A.P., Shadel G.S. (2017). Mitochondrial DNA in innate immune responses and inflammatory pathology. Nat. Rev. Immunol..

[bib81] Westermann B. (2008). Molecular machinery of mitochondrial fusion and fission. J. Biol. Chem..

[bib82] Willner P., Moreau J.L., Nielsen C.K., Papp M., Sluzewska A. (1996). Decreased hedonic responsiveness following chronic mild stress is not secondary to loss of body weight. Physiol. Behav..

[bib83] Willner P., Muscat R., Papp M. (1992). Chronic mild stress-induced anhedonia: a realistic animal model of depression. Neurosci. Biobehav. Rev..

[bib84] Willner P., Towell A., Sampson D., Sophokleous S., Muscat R. (1987). Reduction of sucrose preference by chronic unpredictable mild stress, and its restoration by a tricyclic antidepressant. Psychopharmacology (Berl).

[bib85] Yuan M., Pantazatos S.P., Zhu H., Li Y., Miller J.M., Rubin-Falcone H., Zanderigo F., Ren Z., Yuan C., Lui S., Gong Q. (2019). Altered amygdala subregion-related circuits in treatment-naïve post-traumatic stress disorder comorbid with major depressive disorder. Eur. Neuropsychopharmacol..

[bib86] Zhang F., Rao S., Cao H., Zhang X., Wang Q., Xu Y., Sun J., Wang C., Chen J., Xu X., Zhang N., Tian L., Yuan J., Wang G., Cai L., Xu M., Baranova A. (2022). Genetic evidence suggests posttraumatic stress disorder as a subtype of major depressive disorder. J. Clin. Investig..

[bib87] Zühlsdorff K., López-Cruz L., Dutcher E.G., Jones J.A., Pama C., Sawiak S., Khan S., Milton A.L., Robbins T.W., Bullmore E.T., Dalley J.W. (2023). Sex-dependent effects of early life stress on reinforcement learning and limbic cortico-striatal functional connectivity. Neurobiol. Stress.

