# Supplementary material for: Prelimbic cortex–associated stress sensitisation and region-specific mitochondrial adaptations across preclinical approaches relevant to PTSD and depression
Source: Neurobiol Stress. 2026 Jul 30;44:100840. doi: 10.1016/j.ynstr.2026.100840 (PMC13448202; doi:10.1016/j.ynstr.2026.100840)
Supplement: Supplementary file 1 [file mmc1.pdf]

Supplementary Materials 1

Data-Driven Clustering Approach to Behavioural Phenotyping

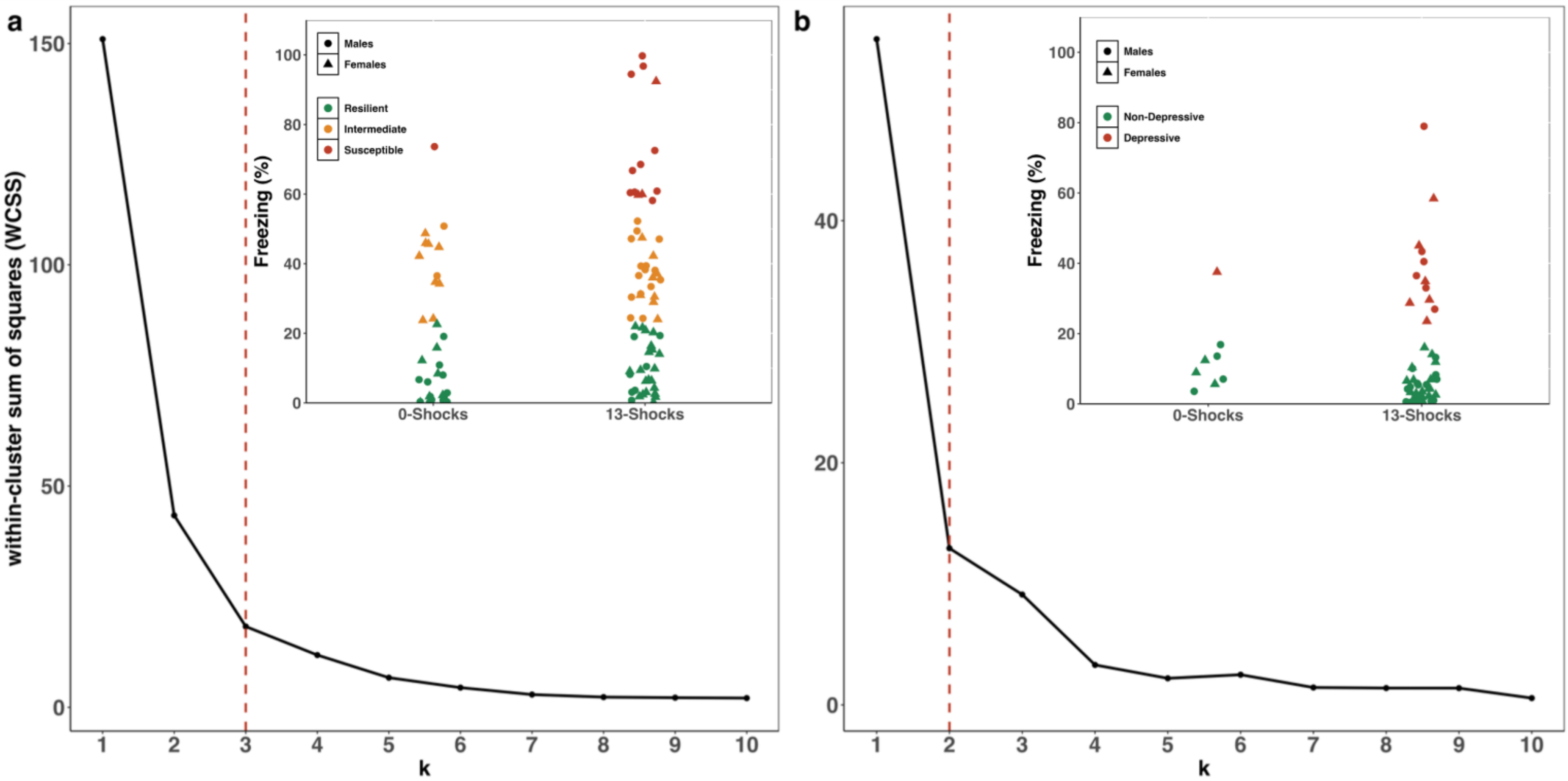

Figure S1.1: Elbow Plot Showing the WCSS for Different Values of K. a) The elbow point, where the rate of decrease in WCSS slowed, indicated that K = 3 was the most appropriate value for clustering the SEFL data. Group sizes: 0-Shocks, n = 32 (Males = 16, Females = 16); 13-Shocks, n = 64 (Males = 32, Females = 32). **b)** The elbow point indicated that K = 2 was the most appropriate value for clustering the chronic stress data. Group sizes: 0-Shocks, n = 8 (Males = 4, Females = 4); 13-Shocks, n = 48 (Males = 24, Females = 24). Inset panel shows the distribution of freezing (mean %) for 0- and 13-Shock animals during the first 8 minutes of the re-exposure session.
