# Supplementary material for: Prelimbic cortex–associated stress sensitisation and region-specific mitochondrial adaptations across preclinical approaches relevant to PTSD and depression
Source: Neurobiol Stress. 2026 Jul 30;44:100840. doi: 10.1016/j.ynstr.2026.100840 (PMC13448202; doi:10.1016/j.ynstr.2026.100840)
Supplement: Supplementary file 2 [file mmc2.pdf]

Oestradiol levels did not influence fear conditioning or extinction in females

Oestrus Checking

Prior to each behavioural session, the oestrus stage of female rats was determined through visual inspection of the external genitalia. This is as accurate and reliable as the vaginal smear whilst being quicker and less invasive (Barret et al., 2010). Given the association between menstrual cycle phase and plasma cytokine levels in humans (O'Brien et al., 2007), oestrus stage was also assessed prior to blood sampling to control for any cyclical variations. Females in proestrus or oestrus were defined as having 'high oestradiol', whilst females in metestrus or dioestrus were classified as 'low oestradiol'.

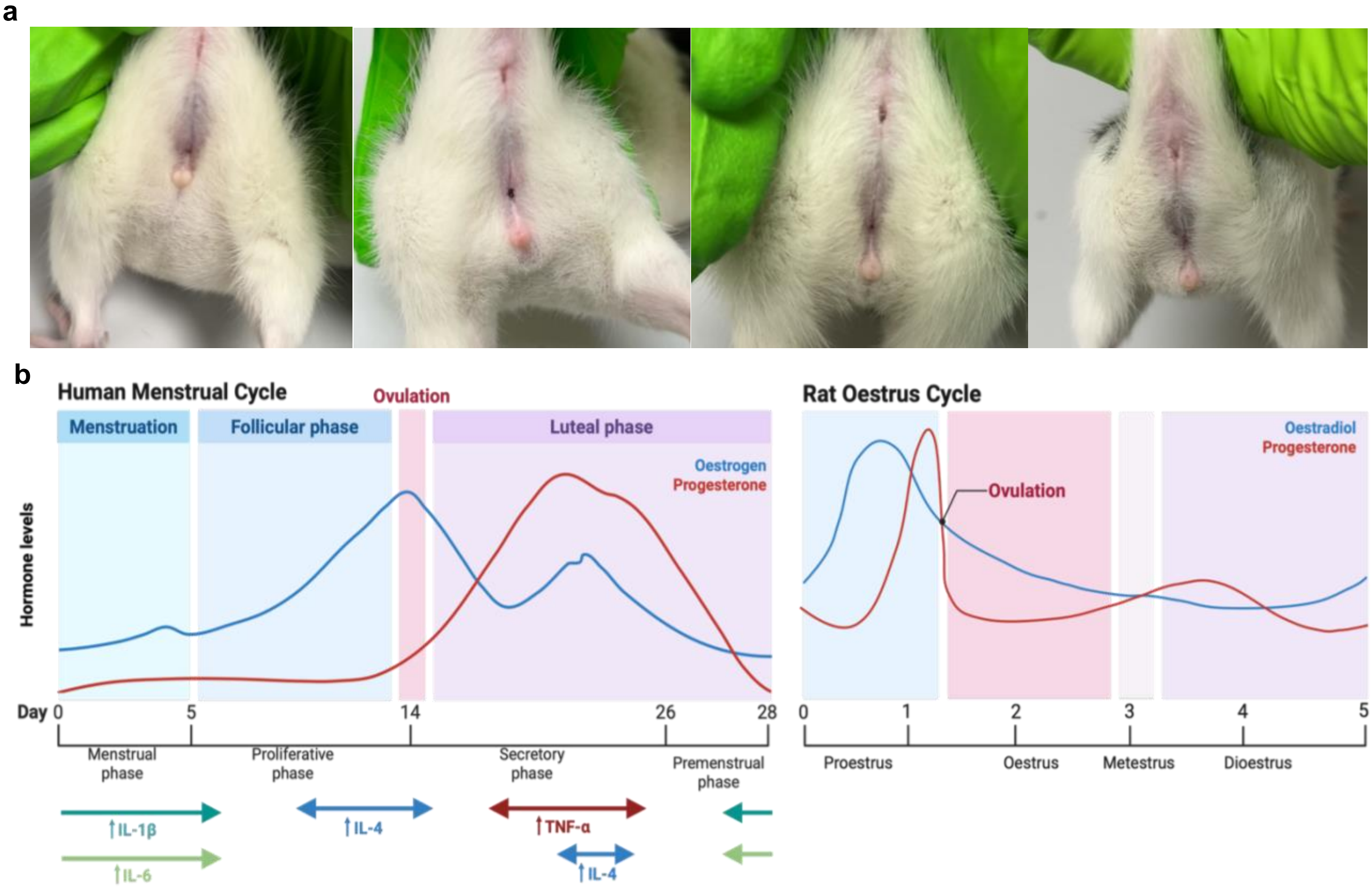

Figure S2.1: Human Menstrual and Rat Oestrus Cycles. The rodent oestrus cycle has four phases (proestrus, oestrus, metestrus and dioestrus) and lasts for 4 to 5 days. In comparison, in humans there are three phases of the menstrual cycle (menstrual, follicular and luteal; (Ajayi & Akhigbe, 2020)). **a)** From left to right: Proestrus: absence of vaginal opening, swollen, pink tissue; Oestrus: distinct vaginal opening; Metestrus: partial vaginal opening; Dioestrus: absence of vaginal opening, no tissue swelling. **b)** The rodent proestrus phase corresponds to the human follicular stage, which is associated with a rise in circulating oestrogen concentrations and a small surge in prolactin, which leads to a rise in luteinising hormone (LH) and follicle-stimulating hormone (FSH) release. The peak in FSH concentration and the associated decline from peak in oestradiol levels correspond to ovulation and the onset of the oestrus phase. Although oestradiol levels begin to fall at this point, they remain relatively elevated compared to the metestrus and dioestrus phases. Metestrus and dioestrus are homologous to human early and late secretory stages of the reproductive cycle, respectively, and are characterised by rising levels of progesterone. Circulating levels of IL-1β and IL-6 are highest during menses when levels of oestrogen and progesterone are low. In comparison, levels of IL-4 show a positive correlation with oestrogen, and levels of TNF-α show a positive correlation with progesterone levels(O'Brien et al., 2007).

Fear Conditioning

Analysis revealed no main effect of oestradiol levels on the relative change in freezing between the 30s prior to the first shock and the 30s following the second shock (Oestradiol: $F_{(1,60)} = 0.65$, $p = .42$, $\eta^2 = 0.006$).

Fear Extinction and Extinction Retention

Oestradiol levels during extinction had no effect on mean freezing during the re-exposure session (Low Oestradiol: $\beta = 0.06$, SE = 1.30, z = 0.04, p = .96, 95% CI [-2.43, 2.55]) or on the rate of extinction learning (Oestradiol: $F_{(1, 56)} = 0.37$, $p = .55$, $\eta^2 = 0.004$). Relative oestradiol levels also did not influence the retention of the extinction memory ($F_{(1,61)} = 0.007$, $p = .93$, $\eta^2 = 0.00009$).

References:

Ajayi, A. F., & Akhigbe, R. E. (2020). Staging of the estrous cycle and induction of estrus in experimental rodents: an update. *Fertility Research and Practice*, *6*(5). https://doi.org/https://doi.org/10.1186/s40738-020-00074-3

Barret, K. E., Barman, S. M., Boitano, S., & Brooks, H. L. (2010). Reproductive development and function of the female reproductive system. In *Ganong's review of medical physiology* (24th editi). McGraw Hill Education.

O'Brien, S. M., Fitzgerald, P., Scully, P., Landers, A., Scott, L. V., & Dinan, T. G. (2007). Impact of gender and menstrual cycle phase on plasma cytokine concentrations. *Neuroimmunomodulation*, *14*(2), 84–90.
