# Supplementary material for: Prelimbic cortex–associated stress sensitisation and region-specific mitochondrial adaptations across preclinical approaches relevant to PTSD and depression
Source: Neurobiol Stress. 2026 Jul 30;44:100840. doi: 10.1016/j.ynstr.2026.100840 (PMC13448202; doi:10.1016/j.ynstr.2026.100840)
Supplement: Supplementary file 3 [file mmc3.pdf]

Supplementary Materials 3

Cytokine ELISA Pools

Pool ID	Stratum	N
1	0_Chronic_Depressive_Female	1
2	0_Chronic_Non-Depressive_Female	3
3	0_Chronic_Non-Depressive_Male	4
4	0_Day 1_Intermediate_Female	3
5	0_Day 1_Intermediate_Male	1
6	0_Day 1_Resilient_Female	5
7	0_Day 1_Resilient_Male	6
8	0_Day 1_Susceptible_Male	1
9	0_Day 13_Intermediate_Female	4
10	0_Day 13_Intermediate_Male	1
11	0_Day 13_Resilient_Female	4
12	0_Day 13_Resilient_Male	4
13	0_Day 13_Resilient_Male	3
14	13_Chronic_Depressive_Female	3
15	13_Chronic_Depressive_Female	3
16	13_Chronic_Depressive_Male	3
17	13_Chronic_Depressive_Male	3
18	13_Chronic_Non-Depressive_Female	6
19	13_Chronic_Non-Depressive_Female	6
20	13_Chronic_Non-Depressive_Female	6
21	13_Chronic_Non-Depressive_Male	5
22	13_Chronic_Non-Depressive_Male	6
23	13_Chronic_Non-Depressive_Male	7
24	13_Day 1_Intermediate_Female	2
25	13_Day 1_Intermediate_Female	3
26	13_Day 1_Intermediate_Male	4
27	13_Day 1_Intermediate_Male	3
28	13_Day 1_Resilient_Female	5
29	13_Day 1_Resilient_Female	4
30	13_Day 1_Resilient_Male	2
31	13_Day 1_Susceptible_Female	2
32	13_Day 1_Susceptible_Male	4
33	13_Day 1_Susceptible_Male	3
34	13_Day 13_Intermediate_Female	2
35	13_Day 13_Intermediate_Male	6
36	13_Day 13_Resilient_Female	6
37	13_Day 13_Resilient_Female	7
38	13_Day 13_Resilient_Male	7
39	13_Day 13_Susceptible_Female	1
40	13_Day 13_Susceptible_Male	3

OXPHOS Western Pools

Pool ID	Stratum	N
1	13_Day 1_Intermediate_Female	5
2	13_Day 1_Intermediate_Male	4
3	13_Day 1_Resilient_Female	5
4	13_Day 1_Resilient_Female	4
5	13_Day 1_Susceptible_Male	3
6	13_Day 1_Intermediate_Male	3
7	13_Day 1_Resilient_Male	2
8	13_Day 1_Susceptible_Male	4
9	13_Day 13_Resilient_Female	4
10	13_Day 13_Resilient_Female	4
11	13_Day 13_Resilient_Female	5
12	13_Day 13_Intermediate_Female	2
13	13_Day 1_Susceptible_Female	2
14	13_Day 13_Intermediate_Male	3
15	13_Day 13_Resilient_Male	3
16	13_Day 13_Susceptible_Male	3
17	13_Chronic_Depressive_Female	3
18	13_Chronic_Non-Depressive_Female	5
19	13_Chronic_Non-Depressive_Female	5
20	13_Chronic_Non-Depressive_Female	5
21	13_Chronic_Non-Depressive_Female	3
22	13_Chronic_Depressive_Male	3
23	13_Chronic_Non-Depressive_Male	5
24	13_Chronic_Non-Depressive_Male	5
25	13_Chronic_Non-Depressive_Male	5
26	13_Chronic_Non-Depressive_Male	3
27	0_Day 1_Intermediate_Female	3
28	0_Day 1_Resilient_Female	5
29	0_Day 1_Intermediate_Male	1
30	0_Day 1_Resilient_Male	6
31	0_Day 1_Susceptible_Male	1
32	0_Day 13_Intermediate_Female	4
33	0_Day 13_Resilient_Female	4
34	0_Day 13_Intermediate_Male	1
35	0_Day 13_Resilient_Male	4
36	0_Day 13_Resilient_Male	3
37	0_Chronic_Depressive_Female	1
38	0_Chronic_Non-Depressive_Female	3
39	0_Chronic_Non-Depressive_Male	4
40	13_Day 13_Intermediate_Male	3
41	13_Day 13_Resilient_Male	4
42	13_Chronic_Depressive_Female	3
43	13_Chronic_Depressive_Male	3
44	13_Day 13_Susceptible_Female	1
