# Supplementary material for: Prelimbic cortex–associated stress sensitisation and region-specific mitochondrial adaptations across preclinical approaches relevant to PTSD and depression
Source: Neurobiol Stress. 2026 Jul 30;44:100840. doi: 10.1016/j.ynstr.2026.100840 (PMC13448202; doi:10.1016/j.ynstr.2026.100840)
Supplement: Supplementary file 4 [file mmc4.pdf]

Molecular Analyses Restricted to Minimal n=3

For ELISA and western blot analyses, in some instances only one biological replicate was available due to pooling and/or the rarity of the phenotype. To avoid a single measurement being taken as representative of a group, we conducted additional analyses restricted to minimal n=3 replicates which are presented below. Whilst the exclusion of low n groups limited our ability to infer changes resulting from prior shock exposure, they did not significantly alter our phenotypic findings.

Plasma Cytokines

TNF-α

Baseline plasma TNF-α concentrations (**Figure S4.1a**) did not differ between males and females (SexFemale: β=0.51, SE=0.13, z=0.41, p=.70, 95% CI [-0.20, 0.30]). However, clear phenotype-related differences emerged: animals later classified as SEFL-Intermediate showed significantly lower baseline TNF-α than both resilient and susceptible groups (resilient > intermediate, p=.003; intermediate < susceptible, p=.03), with no differences between Depressive-Like and Non-Depressive-Like animals or between cohorts (all |β|≤0.0009, p>0.99). At terminal sampling (**Figure S4.1b**), this pattern reversed: intermediate and susceptible animals showed higher TNF-α than resilient rats (PhenotypeIntermediate: β=0.46, SE=0.18, z=2.62, p=.02, 95% CI [0.13, 0.82]; PhenotypeSusceptible: β=0.89, SE=0.12, z=7.13, p=.0002, 95% CI [0.64, 1.13]), forming a graded increase from resilient to intermediate to susceptible. Exclusion of comparisons with n<3 also revealed a difference in terminal TNF-α levels between intermediate and susceptible groups (PhenotypeSusceptible: β=0.80, SE=0.12, z=6.38, p<.0001, 95% CI [0.55, 1.04]) and susceptible and non-depressive-like animals (PhenotypeNon-Depressive-Like: β=-0.79, SE=0.14, z=-5.68, p<.0001, 95% CI [-1.06, -0.52]).
Depressive-Like animals also exhibited elevated TNF-α relative to Non-Depressive-Like animals, although this was smaller when excluding groups of n<3 (PhenotypeDepressive-Like: β=0.54, SE=0.15, z=3.51, p=.002, 95% CI [0.24, 0.84]). No effects of sex, shock history, or cohort were observed at endpoint (all |β|≤0.04, p>0.24).

IL-10

Exclusion of groups with n<3 led to a loss of the previously observed phenotype × shock interaction ($p=.17$). However, the sex difference in Terminal IL-10 levels was retained (**Figure S4.2a)**; $F_{(1,21)}=6.09$, $p=.02$, $\eta^2=0.10$), with females exhibiting higher concentrations than males. Shock-related effects were also detected, with higher levels of this anti-inflammatory cytokine observed in 0-Shock animals ($F_{(1,21)}=14.66$, $p=.0009$, $\eta^2=0.24$).

*IL-1*β

Plasma IL-1β (**Figure S4.2b**) differed by sex ($F_{(1,20)}=5.35$, $p=.03$, $\eta^2=0.12$), with females showed higher circulating IL-1β than males. Exclusion of low n groups from the analysis led to a loss of the previously observed main effect of shock ($p=.34$) but did reveal and interaction between phenotype and prior shock exposure ($F_{(1,20)}=6.42$, $p=.02$, $\eta^2=0.15$), although this did not survive pairwise correction.

IL-6

IL-6 was modulated by shock exposure across stress paradigms ($F_{(1,19)}=39.81$, $p<.0001$, $\eta^2=0.26$), and levels were higher in the CUS cohort ($F_{(1,19)}=45.70$, $p<.0001$, $\eta^2=0.30$); an effect driven by female animals (p.adj<.0001).

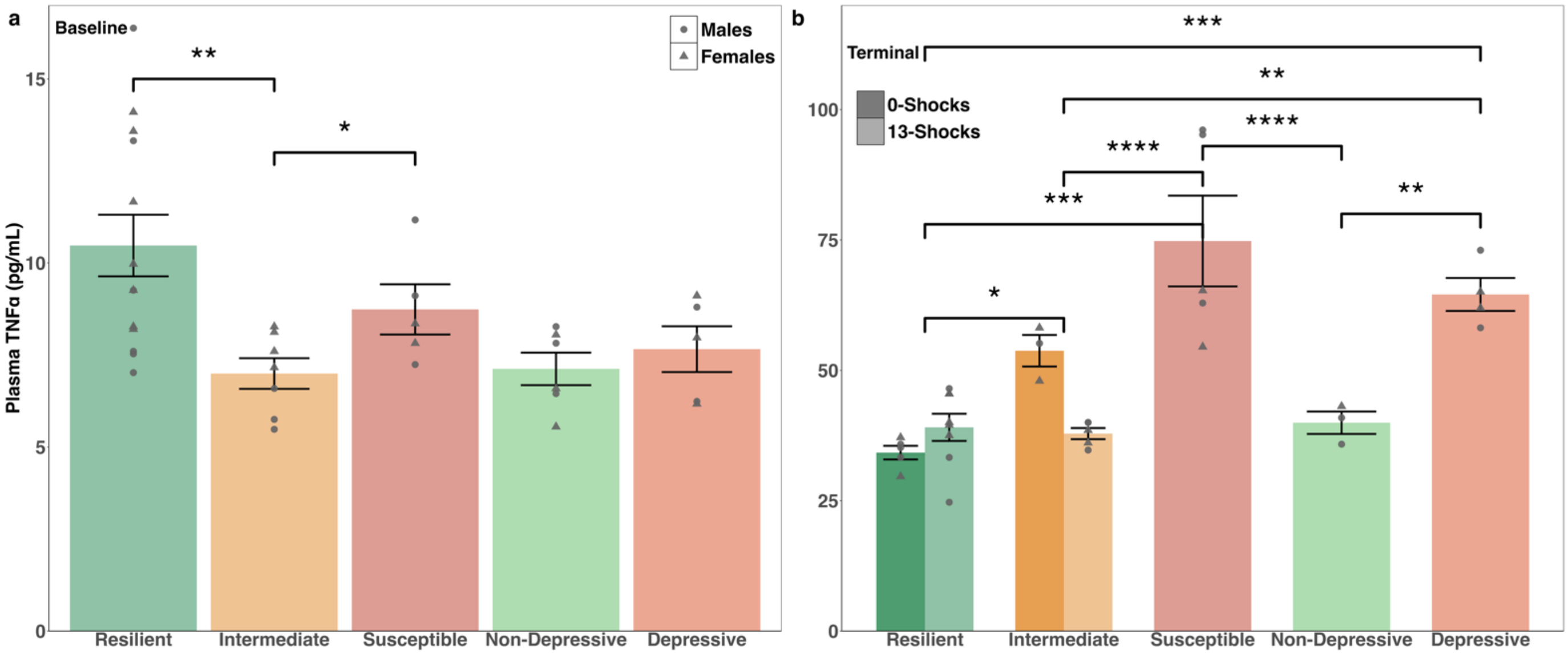

Figure S4.1: Graded elevations in circulating TNF-α concentrations (pg/mL) could be seen across behavioural phenotypes at the terminal timepoint when limiting analyses to minimal n=3. a) Small differences in circulating TNF-α concentrations were observed at baseline in the SEFL cohort. **b)** Terminal TNF-α concentrations increased progressively from resilient < intermediate < susceptible, and from non-depressive-like < depressive-like, reflecting a continuum of inflammatory activation. Data are expressed as mean ± s.e.m.. Individual data points are overlaid. See figure key for colour and transparency mappings used throughout. *p<0.05, **p<0.01, ***p<0.001, ****p<0.0001.

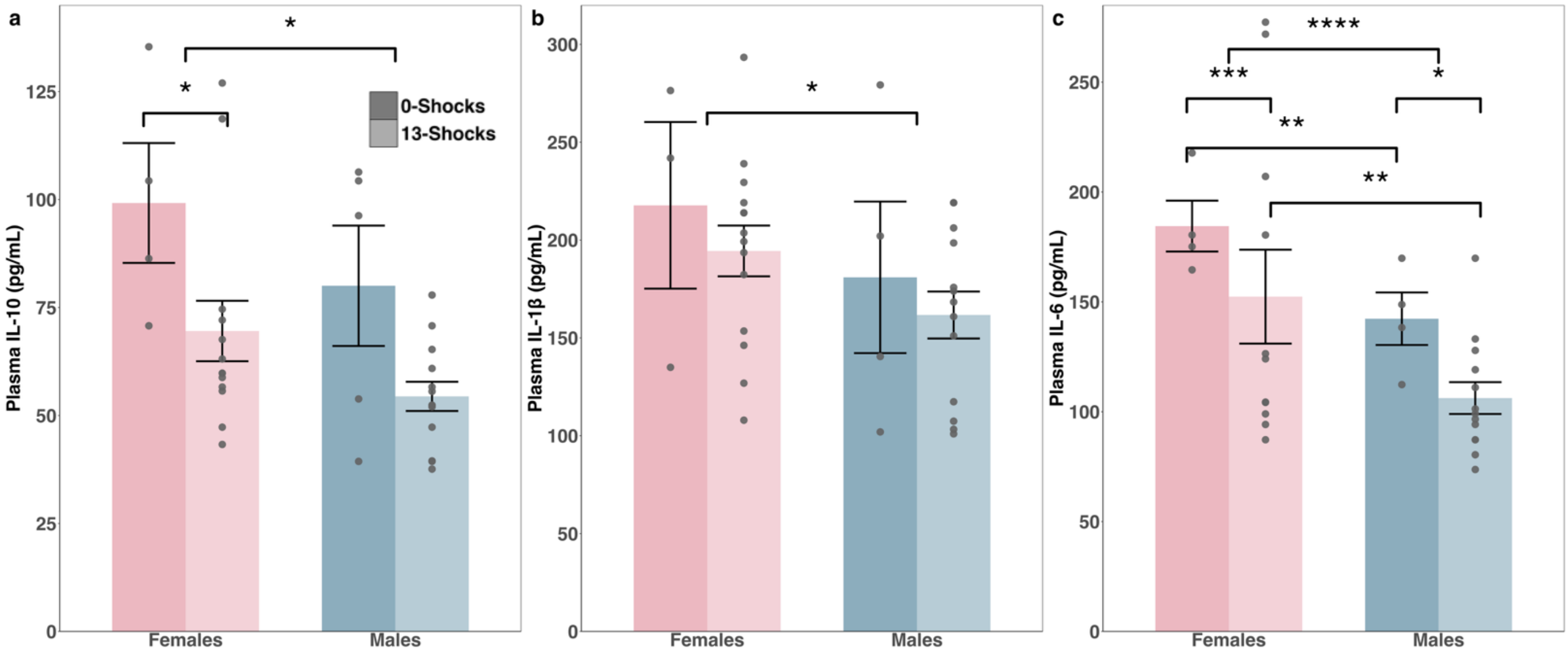

Figure S4.2: Terminal cytokine concentrations (pg/mL). a) Females showed elevated circulating IL-10 at the terminal sampling point**. b)** Females showed elevated circulating IL-1β following behavioural procedures. **c)** Circulating IL-6 was elevated in females compared to males and in 0-Shock animals. Data are expressed as mean ± s.e.m.. Individual data points are overlaid. See figure key for colour and transparency mappings used throughout. *p<0.05, **p<0.01, ***p<0.001, ****p<0.0001.

Oxidative Phosphorylation Markers

Prelimbic Cortex

In the PL of SEFL animals (**Figure S4.3a-b)**, mitochondrial protein expression varied as a function of phenotype (Complex I: $F_{(2,16)}$=4.15, p=.04, η^2=0.27; Complex II: $F_{(2,16)}$=4.65, p=.03, η^2=0.22), with susceptible rats showing reduced Complex I expression relative to resilient animals (p.adj=.05), and Complex II expression differing between resilient and intermediate groups (p.adj=.03), with no effects of shock (p=.76). In contrast, no phenotypic differences were observed for CUS animals (**Figure S4.3k-o)**.

Sex x Shock interactions strongly modulated Complex II and Complex V expression in the SEFL paradigm (Complex II: $F_{(1,16)}$=11.62, p=.004, η^2=0.27; Complex V: $F_{(1,16)}$=4.75, p=.04, η^2=0.14), with females exposed to 13 shocks showed higher CII and CV expression than their male counterparts (**Figure S4.2g and Figure S4.2j**; both p.adj<.04). No sex differences were observed in the CUS cohort when restricting analyses to groups with minimal n=3 and due to low n, shock effects could not be measured for the CUS cohort.

Basolateral Amygdala

In the BLA, Complex V expression was elevated in both SEFL-susceptible (**Figure S4.4e**) and CUS-Depressive-Like (**Figure S4.4j**) animals (SEFL: ($F_{(2,16)}$=7.22, *p*=.006, η^2=0.37; CUS: $F_{(1,7)}$=36.47, *p*=.0005, η^2=0.48) compared to resilient and Non-Depressive-Like groups respectively, contrasting with the PL pattern. Complex IV expression (**Figure S4.4d**) also varied by phenotype in the SEFL cohort ($F_{(2,15)}$=3.54, *p*=.05, η^2=0.20), with some evidence for higher levels in susceptible rats (p.adj=.05). No other phenotype effects were detected (all *p*>.15).

OXPHOS expression showed robust sex differences across both stress paradigms. SEFL males exhibited higher expression of several complexes, with effects most pronounced following 13 shocks. In SEFL animals a significant Shocks × Sex x Phenotype interaction for Complex III expression was observed ($F_{(1,18)}$=7.56, *p*=.01, η^2=0.14), with *post hoc* analyses revealing that sex differences were largest among animals exposed to 13 shocks exhibiting an intermediate phenotype (p.adj=.04). For Complex IV (**Figure S4.4i**), sex differences following SEFL were

observed ($F_{(1,15)}$=9.04, p=.009, η^2=0.25), driven by higher Complex IV expression in males relative to females within the 13-shock condition (p.adj=.05).

In the CUS cohort, males showed higher Compex II (**Figure S4.4q**; $F_{(1,8)}$=15.19, p=.005, η^2=0.56), Complex IV (**Figure S4.4s**; $F_{(1,8)}$=8.71, p=.02, η^2=0.41), and Complex V (**Figure S4.4t**; $F_{(1,7)}$=9.11, p=.02, η^2=0.12) expression.

Infralimbic Cortex

Phenotypic differences in Complex I and Complex V expression within the IL were evident in the SEFL paradigm (CI: $F_{(2,16)}$=7.19, p=.006, η^2=0.43; CV: $F_{(2,15)}$=4.35, p=.03, η^2=0.25). Animals classified as intermediate or susceptible exhibited lower Complex I expression than resilient animals (both p.adj>.03; **Figure S4.5a**). Regarding Complex V expression, pairwise comparisons revealed higher expression in susceptible compared to resilient animals (p=.05; **Figure S4.5e**). A phenotype by sex interaction revealed this difference to be largely driven by female animals ($F_{(2,15)}$=3.82, p=.045, η^2=0.22; intermediate females vs susceptible females p.adj=.03). When excluding low n groups, the previously observed phenotypic difference in Complex I expression following the CUS procedure was no longer significant (p=0.26).

Within the SEFL procedure, a significant effect of prior shock history on the expression of Complex IV emerged ($F_{(1,15)}$=4.97, p=.04, η^2=0.22), with 0-shock animals having higher Complex IV expression in the IL.

Across OXPHOS complexes and stress paradigms, no sex differences were observed in protein expression (all p>.07).

Hippocampus

In the SEFL cohort, mitochondrial protein expression in the hippocampus showed a consistent phenotype-linked increase across multiple complexes even when excluding low n groups (**Figure S4.6a-e**). Susceptible animals exhibited higher expression of Complex I ($F_{(2,16)}$=6.70, p=.008, η^2=0.32), Complex III ($F_{(2,14)}$=5.92, p=.01, η^2=0.29), Complex IV ($F_{(2,16)}$=4.74, p=.02, η^2=0.31), and Complex V ($F_{(2,14)}$=6.84, p=.008, η^2=0.37) relative to resilient or intermediate rats (all p.adj<.04), with Complex II additionally showing a strong phenotype effect ($F_{(2,16)}$=15.69, p=.0002, η^2=0.52) and a Shock × Phenotype interaction ($F_{(1,16)}$=5.71, p=.03, η^2=0.09). For Complex I, there was also a significant Sex x Shock interaction

($F_{(1,16)}$=6.42, *p*=.02, η²=0.15), but no pairwise comparisons met significance (all p.adj<.06).

In the CUS model, no phenotype or sex effects were detected for Complexes I–II (shock effects could not be estimated following exclusion of low n groups). However, the exclusion of low n groups did reveal a previously unobserved phenotypic difference in Complex III expression ($F_{(1,7)}$=7.41, *p*=.03, η²=0.48), with Depressive=Like animals showing elevated expression. Depressive-Like animals also showed elevated Complex IV ($F_{(1,7)}$=20.85, *p*=.003, η²=0.48) and Complex V ($F_{(1,7)}$=10.27, *p*=.01, η²=0.42) expression (**Figure S4.6m-o**), with both complexes additionally exhibiting a Sex × Phenotype interaction (CIV: $F_{(1,7)}$=12.36, *p*=.009, η²=0.28; CV: $F_{(1,7)}$=7.05, *p*=.03, η²=0.29). For Complex V, this interaction was driven by female animals (p.adj>.02).

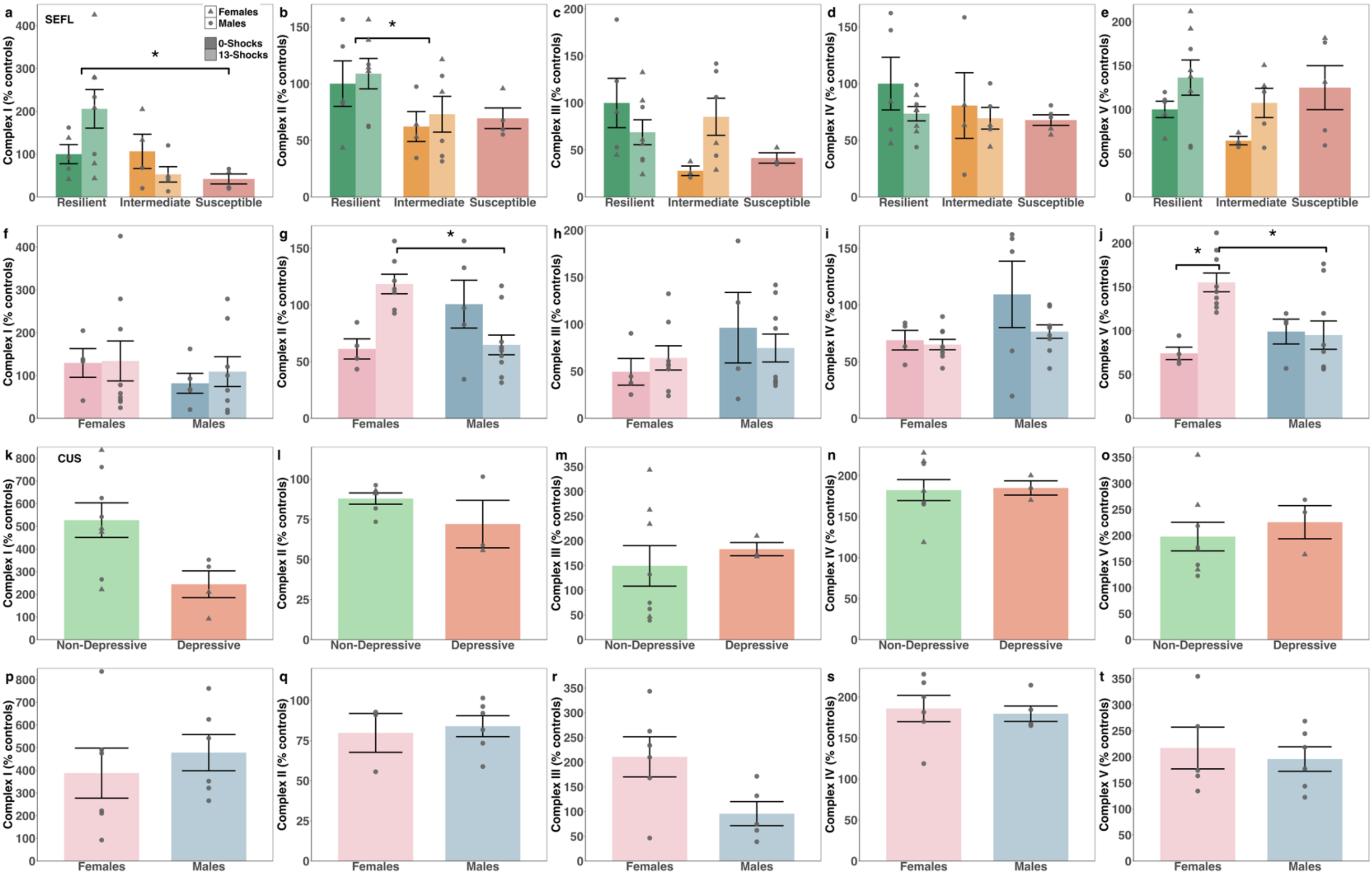

Figure S4.3: Expression of OXPHOS subunits in the PL varied between SEFL phenotypes and sexes, but these differences were not evident in the CUS model. (a-e) In animals assigned to the SEFL procedure, phenotypic variations in CI and CII were observed. Susceptible animals had reduced CI expression and intermediate animals lower CII levels than resilient rats. **(f-j)** CII and CV expression in SEFL rats was elevated in females compared to males. Effects of shock on CV expression were only observed in females, with prior massed shock associated with greater protein expression in the PL of female rats. **(k-o)** No phenotypic differences in OXPHOS subunit expressions were observed in the PL of CUS animals. **(p-t)** No sex differences in OXPHOS subunit expressions were observed in the PL of CUS animals. Data are expressed as mean ± s.e.m.. Individual data points are overlaid. See figure key for colour and transparency mappings used throughout. **p*<0.05, ***p*<0.01, ****p*<0.001, *****p*<0.0001.

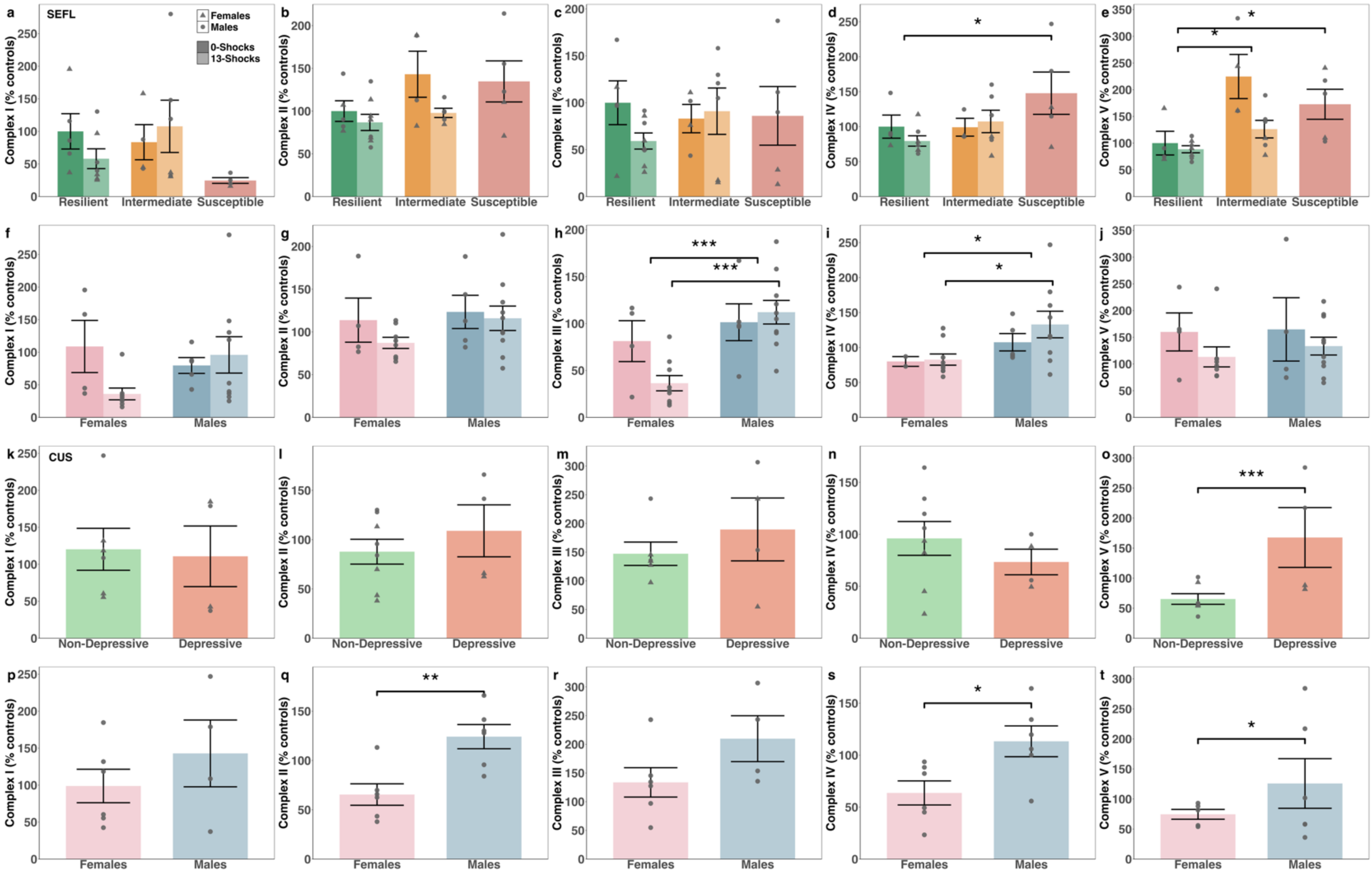

Figure S4.4: Expression of OXPHOS subunits in the BLA varied between phenotypes and sexes in the SEFL paradigm. (a-e) In animals assigned to the SEFL procedure, expression of Complex IV (CIV) was elevated in susceptible animals. Intermediate and susceptible rats had heightened Complex V (CV) expression in the BLA compared to resilient rats. **(f-j)** Complex III (CIII) and CIV expression in SEFL rats was elevated in males, and sex differences were greater in the 13 shock group. **(k-o)** Depressive-like animals showed elevated expression of CV in the BLA compared to their non-depressive-like counterparts. **(p-t)** Males assigned to the CUS procedure showed elevated expression of CII, CIV and CV compared to females. Data are expressed as mean ± s.e.m.. Individual data points are overlaid. See figure key for colour and transparency mappings used throughout. **p*<0.05, ***p*<0.01, ****p*<0.001, *****p*<0.0001.

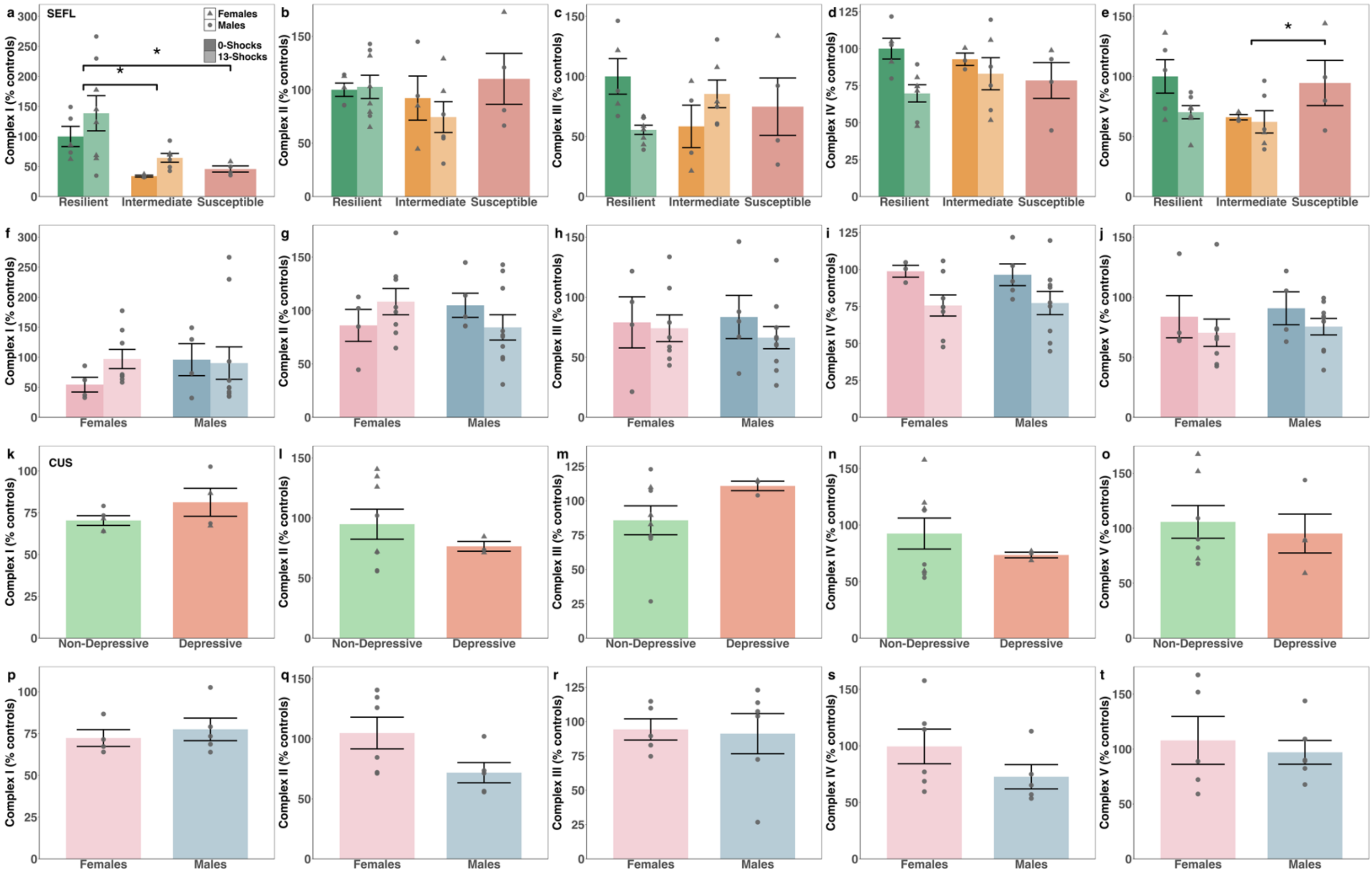

Figure S4.5: Phenotypic differences in OXPHOS subunit expression in the IL were only observed in the SEFL paradigm. (a-e) In animals assigned to the SEFL procedure, expression of Complex I (CI) was decreased in intermediate and susceptible animals; Complex V (CV) expression was elevated in susceptible compared to intermediate animals. **(f-j)** No sex differences in OXPHOS subunit expressions were observed in the IL of SEFL animals. **(k-o)** No phenotypic differences in OXPHOS subunit expressions were observed in the IL of CUS animals. **(p-t)** No sex differences in OXPHOS subunit expressions were observed in the IL of CUS animals. Data are expressed as mean ± s.e.m.. Individual data points are overlaid. See figure key for colour and transparency mappings used throughout. **p*<0.05, ***p*<0.01, ****p*<0.001,

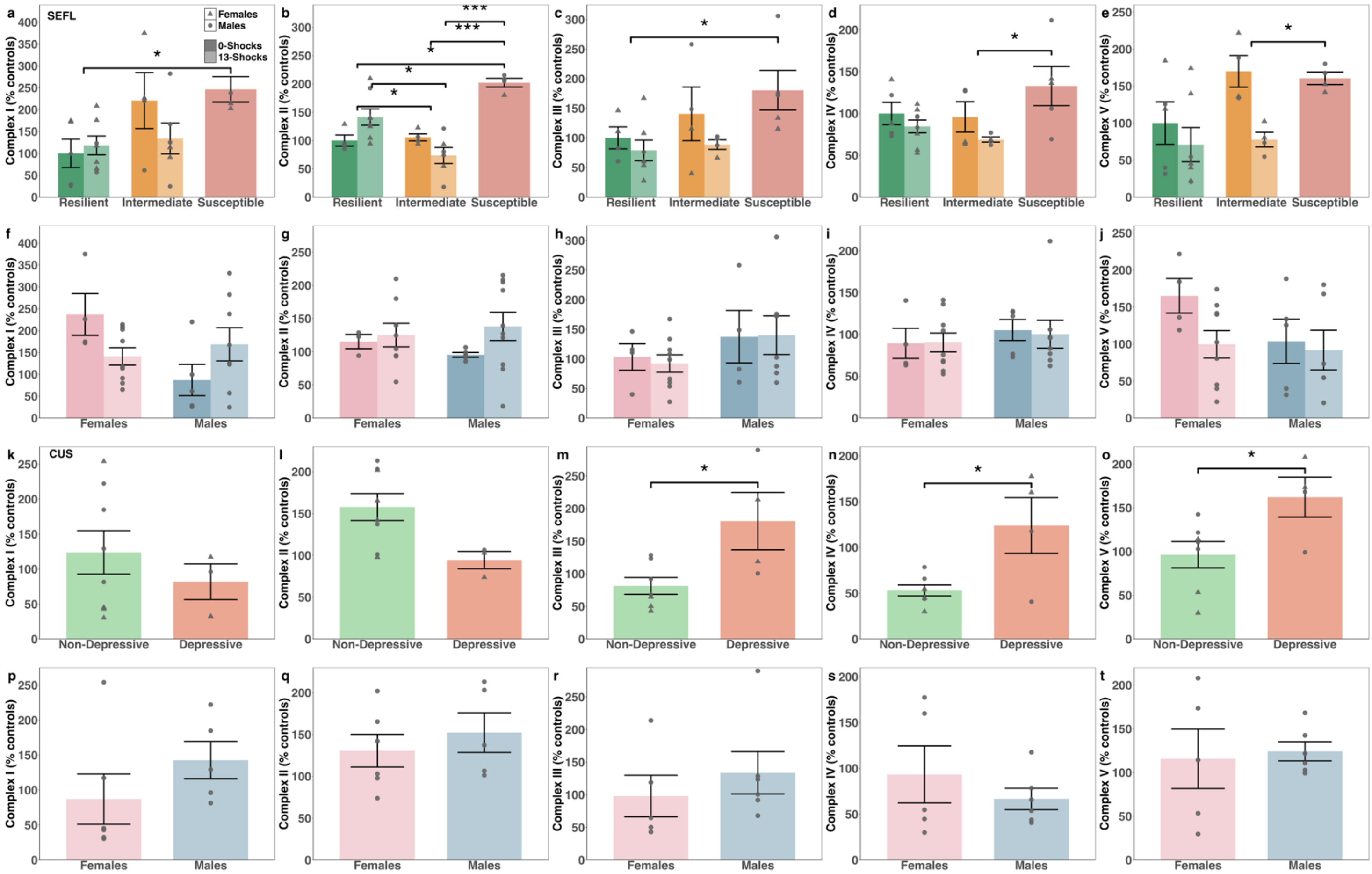

Figure S4.6: Phenotypic differences in OXPHOS subunit expression in the dHpc were observed across stress paradigms. (a-e) In animals assigned to the SEFL procedure, expression of all OXPHOS complexes was elevated in susceptible animals. **(f-j)** No sex differences in OXPHOS subunit expressions were observed in the dHpc of SEFL animals. **(k-o)** Depressive-like animals exhibited increased expression of CIII-V. **(p-t)** No sex differences in OXPHOS subunit expressions were observed in the dHpc of CUS animals. Data are expressed as mean ± s.e.m.. Individual data points are overlaid. See figure key for colour and transparency mappings used throughout. *p<0.05, **p<0.01, ***p<0.001, ****p<0.0001.
