# Supplementary material for: Prelimbic cortex–associated stress sensitisation and region-specific mitochondrial adaptations across preclinical approaches relevant to PTSD and depression
Source: Neurobiol Stress. 2026 Jul 30;44:100840. doi: 10.1016/j.ynstr.2026.100840 (PMC13448202; doi:10.1016/j.ynstr.2026.100840)
Supplement: Supplementary file 5 [file mmc5.pdf]

Supplementary Materials 5

Assessment of TOM70 Expression Across Experimental Groups

To evaluate the suitability of TOM70 as a mitochondrial normalization control, mean TOM70 expression was quantified across all brain regions for each animal and analysed as an outcome variable. A factorial ANOVA was performed with shock exposure, sex, and phenotype as between-subject factors.

No significant main effects of shock exposure ($F_{(1,25)}$ = 0.55, p = .466, η^2 = .01), sex ($F_{(1,25)}$ = 0.73, p = .402, η^2 = .02), or phenotype ($F_{(4,25)}$ = 0.39, p = .813, η^2 = .04) were observed. Furthermore, no significant interactions were detected between shock exposure and sex ($F_{(1,25)}$ = 0.64, p = .432, η^2 = .02), shock exposure and phenotype ($F_{(4,25)}$ = 1.09, p = .381, η^2 = .11), sex and phenotype ($F_{(4,25)}$ = 0.59, p = .676, η^2 = .06), or among shock exposure, sex, and phenotype ($F_{(2,25)}$ = 2.27, p = .124, η^2 = .11).

These findings indicate that TOM70 expression was not systematically influenced by the experimental manipulations or behavioural phenotypes examined in the present study and support its use as a loading control.
