# Supplementary material for: Prelimbic cortex–associated stress sensitisation and region-specific mitochondrial adaptations across preclinical approaches relevant to PTSD and depression
Source: Neurobiol Stress. 2026 Jul 30;44:100840. doi: 10.1016/j.ynstr.2026.100840 (PMC13448202; doi:10.1016/j.ynstr.2026.100840)
Supplement: Supplementary file 6 [file mmc6.pdf]

Analyses indicated no behavioural differences between animals exposed to remote versus recent trauma

Fear Conditioning

Following context B fear conditioning, a significant effect of cohort emerged (Cohort: $F_{(2,124)} = 14.79$, $p < .0010$, $\eta^2 = 0.14$). Pairwise comparison revealed that within the SEFL cohort, the recency of context A ‘trauma’ (remote versus recent) did not influence fear conditioning (p.adj = .14). However, there was a significant difference in the post-shock freezing of the chronic cohort compared to both the remote (p.adj < .0001) and recent (p.adj = .004) trauma groups. Animals assigned to the chronic stress procedure froze significantly less (M = 20.70 ± 1.92%) than animals in both the remote (M = 37.00 ± 3.16 %) and recent (M = 30.30 ± 3.05 %) trauma cohorts.

Fear Extinction and Extinction Retention

No significant differences in mean freezing were observed between the remote and recent trauma cohorts ($\beta = 0.23$, SE = 0.54, z = 0.42, p = .67, 95% CI [-0.83, 1.28]) when accounting for phenotype, shock group, time bin, and sex. No difference in fear recovery was observed between cohorts ($F_{(1,124)} = 0.97$, p = .33, $\eta^2 = 0.007$), and the interaction between phenotype and cohort was non-significant ($F_{(2,124)} = 0.54$, p = .58, $\eta^2 = 0.007$).

Supplementary Materials 6

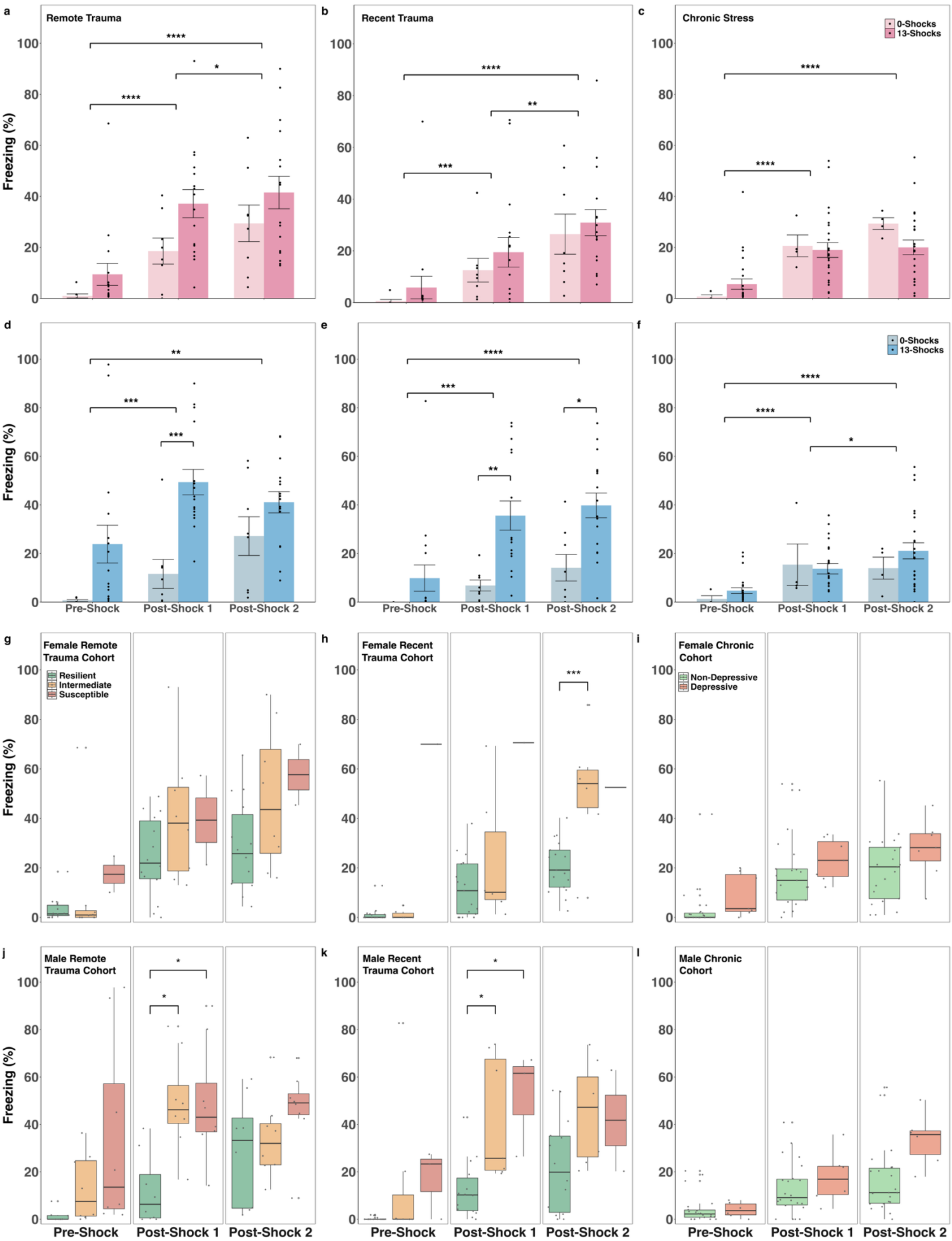

Supplementary Materials 6

Figure S4.1: All animals conditioned fear to context B, but this was modulated by prior shock exposure, phenotype, cohort and sex. a) All females in the remote trauma cohort conditioned fear to context B, and this was not modulated by prior shock exposure. **b)** All females in the recent trauma cohort conditioned fear to context B, and this was not modulated by prior shock exposure. **c)** All females in the chronic stress cohort conditioned fear to context B following the first shock, but no further increase in freezing was observed following the second shock. **d)** Male rats exposed to 13-shocks on day 1 (remote trauma) showed significantly more freezing (%) than 0-shock controls following the first shock **e)** Male rats exposed to 13-shocks on day 13 (recent trauma) showed greater freezing (%) than 0-shock control males after both shocks. **f)** The freezing of males in the chronic cohort was not modulated by prior shock exposure. **g)** No differences between phenotypes were observed in the female, remote trauma cohort during fear conditioning. **h)** A significant difference was observed in the freezing of resilient and intermediate females in the recent trauma cohort following fear conditioning. **i)** No differences between phenotypes were observed in the female, chronic stress cohort during fear conditioning. **j)** A significant difference was observed in the freezing of resilient and both intermediate and susceptible males in the remote trauma cohort following the first shock in context B. **k)** A significant difference was observed in the freezing of resilient and both intermediate and susceptible males in the recent trauma cohort following the first shock in context B. **l)** No differences between phenotypes were observed in the male, remote trauma cohort during fear conditioning. Bar graphs show data expressed as mean ± s.e.m**.** Box plots indicate the median and interquartile range (IQR), with whiskers extending to 1.5×IQR. Individual data points are overlaid. *$p<0.05$, **$p<0.01$, ***$p<0.001$, ****$p<0.0001$.

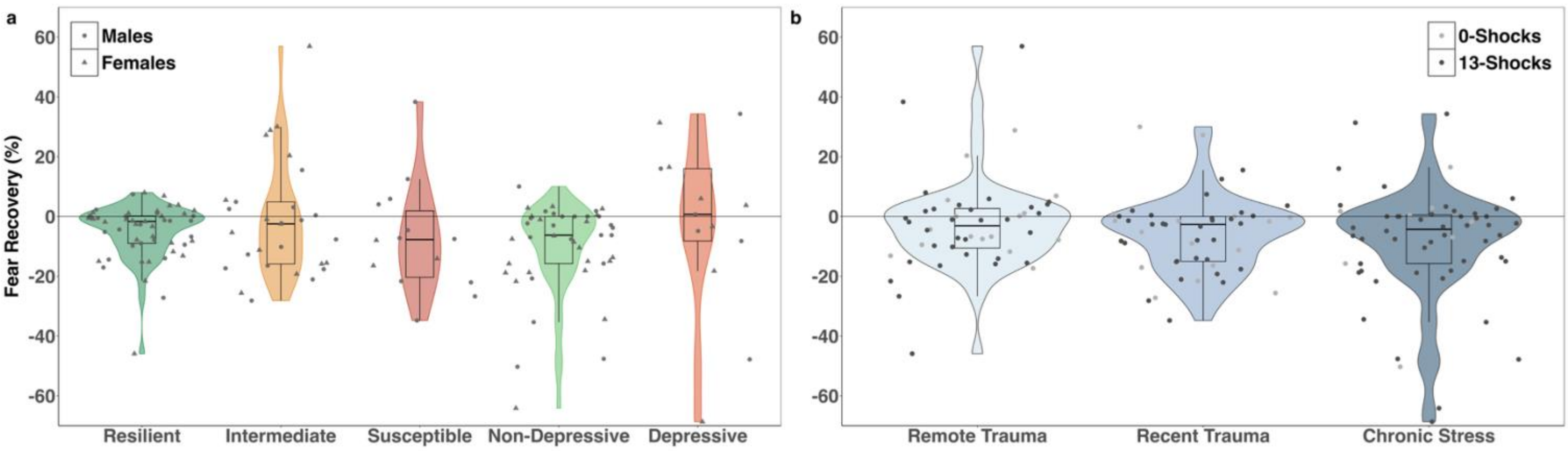

Figure S4.2: Fear recovery (%) did not significantly differ between groups. a) Fear recovery (%) between extinction and test was similar between all phenotypes and did not differ between males and females. **b)** Fear recovery (%) was not influenced by prior shock exposure and did not differ between cohorts. Violin plots show the distribution of the data; box plots indicate the median and interquartile range (IQR), with whiskers extending to 1.5×IQR. Individual data points are overlaid.
